# RelA-Induced Interferon Response Negatively Regulates Proliferation

**DOI:** 10.1371/journal.pone.0140243

**Published:** 2015-10-13

**Authors:** Bose S. Kochupurakkal, Zhigang C. Wang, Tony Hua, Aedin C. Culhane, Scott J. Rodig, Koraljka Rajkovic-Molek, Jean-Bernard Lazaro, Andrea L. Richardson, Debajit K. Biswas, J. Dirk Iglehart

**Affiliations:** 1 Department of Cancer Biology, Dana-Farber Cancer Institute, Boston, Massachusetts, United States of America; 2 Department of Biostatistics and Computational Biology, Dana-Farber Cancer Institute, Boston, Massachusetts, United States of America; 3 Department of Pathology, Brigham and Women's Hospital, Boston, Massachusetts, United States of America; 4 Department of Surgery, Brigham and Women's Hospital, Boston, Massachusetts, United States of America; 5 Department of Cytology, Clinical Hospital Center Rijeka, Rijeka, Croatia; Florida International University, UNITED STATES

## Abstract

Both oncogenic and tumor-suppressor activities are attributed to the Nuclear Factor kappa B (NF-kB) pathway. Moreover, NF-kB may positively or negatively regulate proliferation. The molecular determinants of these opposing roles of NF-kB are unclear. Using primary human mammary epithelial cells (HMEC) as a model, we show that increased RelA levels and consequent increase in basal transcriptional activity of RelA induces IRF1, a target gene. Induced IRF1 upregulates STAT1 and IRF7, and in consort, these factors induce the expression of interferon response genes. Activation of the interferon pathway down-regulates CDK4 and up-regulates p27 resulting in Rb hypo-phosphorylation and cell cycle arrest. Stimulation of HMEC with IFN-γ elicits similar phenotypic and molecular changes suggesting that basal activity of RelA and IFN-γ converge on IRF1 to regulate proliferation. The anti-proliferative RelA-IRF1-CDK4 signaling axis is retained in ER+/HER2- breast tumors analyzed by The Cancer Genome Atlas (TCGA). Using immuno-histochemical analysis of breast tumors, we confirm the negative correlation between RelA levels and proliferation rate in ER+/HER2- breast tumors. These findings attribute an anti-proliferative tumor-suppressor role to basal RelA activity. Inactivation of Rb, down-regulation of RelA or IRF1, or upregulation of CDK4 or IRF2 rescues the RelA-IRF1-CDK4 induced proliferation arrest in HMEC and are points of disruption in aggressive tumors. Activity of the RelA-IRF1-CDK4 axis may explain favorable response to CDK4/6 inhibition observed in patients with ER+ Rb competent tumors.

## Introduction

Five transcription factors RelA, RelB, cRel, NFKB1 (p105/p50) and NFKB2 (p100/p52) constitute the Nuclear factor kappa B (NF-kB) family of transcription factors. All five encode the Rel homology DNA binding domain. In addition RelA, RelB and cRel encodes a trans-activating domain while NFKB1 and NFKB2 encode an Ankyrin repeat region (ARR). Although these five factors are capable of forming all combinations of homo- and hetero-dimers, the predominant pairing occurs between RelA/cRel with the processed form of NFKB1 (p50, lacking the ARR) and RelB with NFKB2. Inactive RelA/cRel-p50 complex resides in the cytoplasm bound to Inhibitor of kappa B (IkB) proteins that encode an ARR while RelB-NFKB2 is restricted to the cytoplasm by the ARR of NFKB2. Activation of the NF-kB pathway occurs through the canonical and non-canonical pathways involving distinct kinase complexes and cell surface receptors. The canonical pathway involves an Inhibitor of KappaB kinase (IKK/IKBK) complex comprising IKK-α, IKK-β and IKK-γ. When activated, the kinases phosphorylate IkB bound to RelA/cRel-p50 and targets IkB for ubiquitination and degradation allowing RelA/cRel-p50 to translocate to the nucleus and regulate gene expression. The non-canonical pathway involves NF-kB inducing kinase (NIK/MAP3K14) upstream of an effector kinase complex formed by a homo-dimer of IKK-α. NIK phosphorylates IKK- α which in-turn phosphorylates residues in the C-terminus of p100 resulting in removal of the ARR domain, translocation of the RelB-p52 complex to the nucleus and regulation of target genes. NF-kB target genes are implicated in developmental programs, cellular response to pathogens, metabolic and genotoxic stress, suppressing apoptosis and regulating proliferation in multiple tissues including the mammary gland. [1, 2].

Inhibiting NF-kB activation in the murine mammary gland by expressing a dominant-active IkB- α or inactivating IKK- α delayed morphogenesis, retarded formation of murine tumors driven by *c-erbB2*/HER2/*neu* (HER2) and Polyoma middle T antigen, and decreased susceptibility to carcinogen induced tumors [3–5]. On the contrary, knocking down IkB- α or overexpression of cRel caused mammary epithelial hyperplasia and tumors [6, 7]. Although murine cells are transformed by fewer oncogenes and the biology of the murine mammary gland is different from the human, these studies incriminate NF-kB in tumorigenesis [8, 9].

Human breast cancer is a heterogeneous disease and may be subdivided based on the expression of estrogen receptor (ER, ER-positive, ER+; ER-negative, ER-), HER2 and pathologic grade: Luminal A (ER+, low grade), Luminal B (ER+, higher grade), HER2-positive (either ER+ or ER-) and Basal-like (ER-/HER2-) [10]. NF-kB activation is higher in ER- breast cancer cell lines and tumors, and NF-kB activation negatively correlated with ER content [11–15]. Using nuclear staining as a surrogate for activation, immuno-histochemical (IHC) analysis of breast tumors found NF-kB activation correlated with ER-negativity, pathological grade, and higher Ki67 [16]. Inhibiting NF-kB sensitize breast cancer cell lines to apoptosis both in-vitro and in xenograft models and activation confers resistance to chemotherapy and targeted agents [17].

Nearly 26% of breast tumors analyzed by The Cancer Genome Atlas (TCGA) have genomic alterations in at least one of the core NF-kB pathway genes; these alterations are present across all subtypes of breast tumors. Most prominent are alterations in upstream kinases IKK-β and IKK-ε, which are amplified, while copy number loss occurs in CYLD, a negative regulator. In fact, these genomic alterations appear to be enriched in the luminal A and luminal B subtypes (S1 Fig). Although there may be an excess of active NF-kB in high-grade proliferative breast tumors, as measured by IHC, the presence and effect of NF-kB in ER+ cancers is less appreciated.

In contrast, a tumor-suppressive role for NF-kB has been observed in different cell types. Overexpression of RelA in a breast cancer cell line or pro-B cells, and expressing cRel in HELA cells and RelB in murine fibroblasts retards proliferation [18–20]. RelA^-/-^ mouse embryonic fibroblasts escape senescence faster, accumulate genomic aberrations and exhibit a transformed phenotype [21]. NF-kB regulates expression of genes associated with the senescence associated secretory phenotype [22]. Furthermore, RelA was the predominant factor bound to chromatin in oncogene-induced senescent cells [23]. In the stratified epithelium of human and mouse skin, RelA translocates to the nucleus coincident with growth arrest towards the outer epithelial layers [24]. There are differing effects of NF-kB in general, and RelA in particular. The molecular determinants governing opposing tumor promoting and inhibitory activities of RelA is unclear.

To evaluate the role of RelA in cell proliferation, we chose primary human mammary epithelial cells (HMEC) and a strategy to allow conditional expression of RelA. Elevation of RelA levels in primary epithelial cells caused proliferation arrest. Mechanistic studies identified activation of the interferon pathway through interferon response factor 1 (IRF1) a target of RelA. The interferon response suppressed cyclin-dependent kinase 4 (CDK4) expression resulting in hypo-phosphorylation of Retinoblastoma (Rb) and proliferation arrest. Analysis of TCGA data confirmed retention of the RelA-IRF1-CDK4 axis among the ER+/HER2- subtype of breast cancers. Finally, using a semi-quantitative IHC assay to assess the intracellular distribution and relative levels of RelA in breast tumor sections and Ki67 as a marker for proliferation, we confirmed the negative correlation between elevated RelA and proliferation in ER+/HER2- tumors.

## Materials and Methods

### Chemicals, Cell lines and Antibodies

All chemicals were obtained from either Sigma-Aldrich (St. Louis, MO) or Thermo Fisher (Waltham, MA) unless specified otherwise. All cell culture media were sourced from Life Technologies (Carlsbad, CA), Fetal Bovine Serum from Clontech Laboratories (Mountain View, CA) and plastic ware from BD (San Jose, CA). HEK293 cells were from ATCC (Manassas, VA), Phoenix cells for retroviral production were from Orbigen (San Diego, CA), hTERT immortalized p16-negative Human Mammary Epithelial Cells (HMEC) were a gift from Prof. Jean Zhao [25], HTERT and Tp53-R175H immortalized Fallopian Tube epithelial cells (FT282) were a gift from Prof. Ronny Drapkin [26]. Anti-RelA (SC-8008, Santa Cruz Biotechnology, Dallas, Texas) was used for IHC and Western blots. Other antibodies used for Western blots were Anti-RelB (SC-226), anti-cRel (SC-71), anti-IkB-alpha (SC-371), anti-CDK4 (SC-260), anti-CDK6 (SC-177), anti-CyclinD1 (SC-246), anti-Actin (SC-1616), anti-HSP90 (SC-1055), anti-IRF2 (SC-498) all from Santa Cruz Biotechnology, anti-Rb (Ab-5) and anti-p53 (Ab-6) from Calbiochem (EMD Millipore, Bellerica, MA). Antibodies to p27, phospho-Rb, RelA (S536), RelA (S468), RelA (Ac-K310), phospho-ERK, phospho-AKT, IRF1, STAT1, IRF6, STAT2, IRF7 and PARP were from Cell Signaling (Danvers, MA). Anti-GFP (JL-18) was from Clontech Laboratories. Anti-Flag antibody (FLAG-M2) was from Sigma-Aldrich.

### Plasmids

The pRetro-X-Tet two-plasmid TET-ON system (pRXTP, Clontech Laboratories) was used in the study. The ORFs of RelA, RelB, cRel, and IRF2 were obtained from Prof. Mark Vidal (The CCSB ORFEOME collection) and CDK4 from Jean-Bernard Lazaro [27]. These ORFs were subcloned into pRXTP (puromycin resistance, Clontech Laboratories) or the pRXTN (Nourseothricin selection) [28]. The pRXTSN plasmid was generated by cloning the mir155 flanking sequences into the pRXTN plasmid at BamH1 and EcoR1 sites, an adaptation of the Block-iT Pol II miR RNAi Expression vectors (Life Technologies, K4935-00) (S4A Fig). Sequences used to generate miRsh-RNA plasmids are listed below. To select optimal miRsh-RNA sequences, we scanned literature and curated shRNA databases and identified shRNAs that were validated previously for knock-down efficiency [29]. The sequences of all miRsh RNAs used in this study are given in Table 1.

**Table 1:** Sequence of indicated miRsh RNAs used in this study.

miRsh-Control	TGCTGAAATGTACTGCGCGTGGAGACGTTTTGGCCACTGACTGACGTCTCCACGCAGTACATTT
miRsh-CDK4	TGCTGTAGTGTAGAGAAATGGGAAGGGTTTTGGCCACTGACTGACCCTTCCCATCTCTACACTA
miRsh-Rb	TGCTGAAATAATGTGGCTTTGAACATGTTTTGGCCACTGACTGACATGTTCAACCACATTATTT
miRsh-IRF1	TGCTGTAAAGTCTCCATAGACAGAGGTGTTTTGGCCACTGACTGACACCTCTGTATGGAGACTTTA

The pWZL-Blast-SV40LT and pWZL-Blast-DDp53 plasmids were gifts from Prof. James DiCaprio and Prof. Jean Zhao respectively [30, 31]. The ORF for RelA mutant that binds IkB-alpha weakly (RelA-CA) was a Gift from Prof. Harikrishna Nakshatri [32]. GFP-RelA and GFP-RelA-CA were cloned into pQCXIP a retroviral vector (Clontech Laboratories). Lentiviral shRNA vector TRCN0000014684 was used to stably knock-down RelA [33]. All constructs were sequenced using Sanger sequencing and accuracy was ensured by comparing the sequence to the Consensus Coding Sequence of each gene [34].

### Generation of HMEC and FT282 derivatives expressing ectopic genes under the control of the Tetracycline promoter

Human Telomerase immortalized (Hygromycin selection) post-stasis p16-negative Human Mammary Epithelial Cells (HMECs) were a gift from Prof. Jean Zhao [25, 35]. Telomerase (Hygromycin selection) and Tp53-R175H (Blasticidin selection) immortalized FT282 cells were a gift from Prof. Ronny Drapkin [26]. HMEC cells were grown in DMEM-F12 supplemented with Insulin (Life Technologies, 10ug/ml), Epidermal Growth Factor (EGF, Peprotech, 10ng/ml), Cholera toxin (Sigma Aldrich, 10ng/ml), Hydrocortisone (Sigma Aldrich, 500ng/ml), Fetal Bovine Serum (FBS Clontech Laboratories, 0.6%) and PenStrep (Life Technologies). FT282 cells were cultured as described previously [26]. Production of retroviruses and infection of HMEC and FT282 cells were performed using standard protocols. First, HMEC and FT282 cell line derivatives harboring the Tet-trans-activator was generated (G418 selection, 400ug/ml). Next derivatives of these cells harboring RelA, RelB or cRel were generated (HRA, HRB, HRC respectively, Puromycin, 1ug/ml). HT cells harboring pRXTSN-miRsh-CDK4 (Hkd-CDK4) or pRXTSN-miRsh-Control (Hkd-C), and HRA cells harboring pRXTSN-miRsh-Rb (HRA-kd-Rb), pRXTN-miRsh-IRF1 (HRA-kd-IRF1), pRXTN-CDK4 (HRA-CDK4) or pRXTN-IRF2 (HRA-IRF2) were generated using Nourseothricin selection (100ug/ml). HRAD and HRA-SV40LT cells were generated by infecting HRA cells with retroviruses generated from pWZL-Blast-DDp53 plasmid or pWZL-Blast-SV40LT (selected using Blasticidin, 2.5ug/ml). RelA knockdown was performed using Lentiviral construct encoding shRNA to GFP or RelA (TRC shRNA library) [36]. The cell lines used in the study are shown in Table 2.

**Table 2:** List of all cell lines used in this study.

Initial cell line	Plasmid introduced	Resultant cell line
HMEC	Tet-trans-activator	HT
HT	pRXTP-RelA	HRA
HT	pRXTP-RelB	HRB
HT	pRXTP-cRel	HRC
HRA	pWZL-Blast-DDp53	HRAD
FT282	Tet-trans-activator	FT
FT	pRXTP-RelA	FRA
HRA	pWZL-Blast-SV40st	HRA-st
HRA	pWZL-Blast-SV40LT	HRA-SV40LT
HRA	pRXTSN-miRshRB1	HRA-kd-Rb
HT	pRXTSN-miRshControl	Hkd-C
HT	pRXTSN-miRshCDK4	Hkd-CDK4
HRA	pRXTSN-miRshIRF1	HRA-kd-IRF1
HRA	pRXTN-IRF2	HRA-IRF2
HMEC	pLKO-shGFP	Sh-GFP
HMEC	pLKO-shRelA	Sh-RelA
HEK293	pQCXIP-GFP-RelA	HERA
HEK293	pQCXIP-GFP-RelAc	HERAc
HEK293	Plko-shRelA	HEkd-A

### Proliferation assay

HMEC (20,000 cells/ml) or FT282 (10,000 cells/ml) cell derivatives were plated in 96 well plates (100ul/well) in triplicates. One day post plating, the cells were treated +/- Dox (0.1 or 1ug/ml) and MTS assays was performed on Days 0 (day Dox was added), 1, 2 and 3 post Dox addition to estimate the amount of viable cells using prescribed protocol (Promega, Madison, WI). MTS values obtained for Day 1, 2 and 3 were normalized to the amount of cells on Day 0 and plotted as fold increase in growth.

### Mammosphere formation assay

HRA and HRAD cells were trypsinized counted and suspended in complete medium supplemented with B27 supplement (1:50, Life Technologies) and Methocult medium (Stem Cell Technology, Vancover, Canada) final 1.6% methyl cellulose) at a density of 10,000 cells/ml. This suspension was carefully layered over a layer of 0.66% Agar in complete growth medium in 6 well tissue culture plates (BD Bioscience). The cultures were monitored on alternative days and pictures were taken two weeks post plating.

### Preparation of samples for Flow cytometry, western blot and gene expression analysis

HMEC cells are sensitive to contact inhibition at high density and plating them at densities less than 20,000 cells/ml drastically reduces viability. To maintain logarithmic growth over 96 hour time-course of the experiment, the cells were plated at ~ 50,000 cells/ml in 6-well plates. The cells were treated with Dox 24 hours post plating according to the scheme described in the supplementary figure. To maintain uniformity in Dox withdrawal experiments, media in all samples was replace irrespective of change in conditions (Dox addition or withdrawal) at particular time points. For cell cycle distribution analysis by flow cytometry, cells were trypsinized, washed and single cell suspensions were fixed in 70% ethanol and stained using Propidium Iodite (Cell Signaling Technology). Samples were analyzed on a BD Fortessa instrument (BD Biosciences). For analysis of whole cell extracts by Western blot, cells were washed with cold PBS, and lysed using a buffer containing 10mM HEPES pH 8.0, 8M Urea, 1% CHAPS, 150 mM NaCl, 1mM EDTA, 10mM glycine supplemented with phosphatase and protease inhibitors (Roche Lifesciences, Indianapolis, IN). Samples ordering for Western blots in the manuscript were performed as shown in figure panels. IR-dye secondary antibodies (Licor) were used for detection and the images were captured using the Licor scanner with intensity settings set to “Auto”. For gene expression analysis, the cells were washed in cold PBS, lysed using Trizol and total RNA was isolated using the miRNA easy kit (Qiagen, Valencia, CA).

### Dox induction, growth factor and interferon stimulation, nucleo-cytoplasmic extraction, NF-kB luciferase reporter and drug sensitivity experiments

Dox induction experiments were performed by plating cells at a density of 50,000 cells/6 well plate and switching the media to Dox containing media 12 hours later. Western blots for Dox induction experiments were performed 48 hours after induction. The time course experiments were performed by plating cells and treating them with DOX 12 hours later. The cells were harvested in lysis buffer at each time point. For the growth factor stimulation experiment, HRA cells were plated and 12 hours later switched to medium with or without Dox and devoid of supplements. After 24 hours, the cells were stimulated with supplement free medium (SM), full medium (FM), SM + EGF (10ng/ml) or Insulin (INS; 10 μg/ml) for 15 minutes. Post stimulation, the cells were placed on ice and processed for Western blotting. For the interferon stimulation experiment, shGFP and sh-RelA cells were plated in 6 well plates and stimulated with IFN-γ (10ng/ml) or IFN-λ1 (10ng/ml) for 48 hours, 12 hours post plating. The samples were processed by western blot. HRA cells were treated with Dox for 48 hours after 12 hours after plating. The cells were washed in cold PBS, washed 1X with Hypotonic buffer (10mM HEPES (pH 7.9), 1.5mM MgCl2 and 10mM KCl) and rocked for 10 min in a fresh aliquot of hypotonic buffer on ice. Cells were harvested by scraping, passed through a 27 gauge needle (10 strokes, cell disruption was monitored under the microscope). The lysate (cytoplasmic extract containing intact nuclei) was spun at 14000 rpm for 10 min. The supernatant was the cytoplasmic extract. The pellet was washed once with hypotonic buffer and the resulting pellet (nuclear extract) was lysed in lysis buffer. A 1:5 (cytoplasm: nucleus) ratio of total protein in each extract was used in Western blots. To perform the NF-kB luciferase reporter experiment, HRA cells were plated and co-transfected with pNF-kB-luc (Clontech Laboratories) and pRL-TK (Promega Corporation). After 12 hours, the cells were plated in triplicates and Dox was added to the cultures after another 12 hours for 48 hours. Firefly and Renilla luciferase activity in the lysates was determined using the Dual-Luciferase Reporter Assay System (Promega Corporation) according to manufacturer’s protocol. To analyze the effect of CDK4/6 and CDK2 inhibitor on CDK4 knock-down cells, Hkd-CDK4 cells were plated at 50,000 cells/well in a 6 well plate and 12 hours post plating, Dox (1μg/ml) was added to induce miR-shCDK4 expression. After 24 hours, the cells were incubated with the CDK4/6 inhibitor PD0332991 (200nM, Selleckchem, Houston, TX) or the CDK2 inhibitor CVT-313 (5μM, Santa Cruz Biotechnology) for 12 hours. Whole cell lysates were prepared and processed for Western blotting.

### HRA Gene expression analysis

Samples for gene expression analysis was generated as described in the previous section. The Affymetrix Primeview array (Santa Clara, CA) was used to generate gene expression data according to manufacturer’s protocol at the Molecular Biology Core Facilities at Dana-Farber Cancer Institute. Probe summarization was performed using the Brainarray ENSG V18 Chip Definition File and RMA normalized using the GenePattern web server at the Broad Institute [37, 38]. GSEA analysis for enrichment of transcription factor target genes was performed on the GenePattern server using the C3: motif gene sets from MsigDB [39]. Differentially expressed genes were identified using Limma implemented in MEV [40]. Hierarchical clustering and generation of heatmaps were performed using GenePattern. The gene expression data can be accessed at Gene Expression Omnibus (GEO) using accession GSE65040.

### Analysis of gene expression data from TCGA samples

Gene expression values for TCGA samples were obtained from Firehose at Broad Institute [41]. Tumor purity estimate for the samples was obtained from the ESTIMATE database [42]. The analysis was restricted to ER+/HER2- tumors. The Kendall-Tau correlation between gene expression and tumor purity was estimated in R. We observed a strong effect of tumor purity on gene expression. At a tumor purity > 75%, the correlation between expression of genes used in the analysis and tumor purity became insignificant (Kendall-Tau correlation coefficient range between -0.2 to + 0.2) while retaining sufficient number of tumors for analysis (n = 75).

### Development of a semi-quantitative IHC protocol for RelA

Stable HEK293 cell derivatives expressing GFP-RelA (HERA) or GFP-RelAc (HERAc) were generated by retroviral transduction and selection using Puromycin (1μg/ml). RelAc is a constitutively active mutant of RelA with low affinity for IkB-α and is predominantly nuclear [32]. A screen, using commercially available anti-RelA antibodies was performed and co-localization of the GFP and secondary Donkey anti-mouse/rabbit-Cy5 signals were sought. This screen identified SC-8008 (monoclonal, Santa-Cruz Biotechnology) as the optimal monoclonal antibody for detecting RelA both in the cytoplasm and nucleus. Next we generated paraffin blocks of HERA, HERAc, HEK293 and HEkd-A (HEK293 cells where RelA was knocked down) cells. A second screen was performed to determine optimal antigen retrieval conditions and antibody dilution for semi-quantitative staining of RelA in 4uM sections of tumor tissue embedded in paraffin. An anti-GFP antibody for which staining conditions were previously standardized was used as a control to further confirm specificity. These screens identified a protocol with the following conditions: **i)** De-paraffinization using standard procedure, **ii)** Antigen retrieval using antigen retrieval solution pH 6.0, (DAKO, Carpenteria, CA) in a pressure chamber for 20 min, iii) Incubation with SC-8008 (1:1400) in Antibody dilution buffer (DAKO) for 1hr at room temperature, **iv)** Wash 3X with phosphate buffered saline containing 0.05% Tween-20 (PBS-T), **v)** Incubation with secondary polymer linked anti-mouse-HRP (DAKO; 1:1000) dilution for 1 hour, vi) Wash 3X with PBS-T, **vii)** Hematoxylin staining, viii) Mount slide. The dilution factor for SC-8008 was lot specific and each lot had to be standardized using the paraffin sections of HEK293-derivatives for optimal results. Paraffin blocks containing SKOV3 cells stimulated with or without TNF-α for 15 minutes were stained using the described staining protocol and detection of both cytoplasmic and nuclear fractions of RelA was verified.

### Tissue Microarray staining and analysis

Two Tissue Microarrays (TMA) i) Boston-cohort and ii) Croatia-cohort were used in the study. Samples in the Boston-cohort comes from patients at DF/HCC and described previously [43]. Procurement of samples in the Croatia-cohort was described previously [44]. Staining intensity and RelA distribution in the Boston-cohort was scored by a pathologist. Cytoplasmic RelA staining intensity scores were cohort specific and was scored from 0–3 (DFCI-cohort) and 1–3 (Croatia-cohort). Nuclear scores for the DFCI-cohort was assigned based on presence of positive nuclei in multiple 40X fields. No evidence of nuclear positivity was scored “0”, samples with rare positive nuclei were scored “1”, score “2” was assigned to samples with more than one field with positive nuclei while samples where almost all fields contained positive nuclei were scored “3”. Scores obtained with the Boston-cohort was dichotomized 0 and 1, and, 2 and 3 were combined. In the Croatia cohort, cytoplasmic score 1 = low, 2 and 3 = high, Nuclear scores were dichotomized at 10% of cells with nuclear staining. Statistical analysis of the staining data was performed using STATA.

### Statistical analysis

For proliferation and luciferase reporter assays, data analysis was performed in MS-Excel. MTS and Luminescence values of the Dox induced samples were normalized to the respective un-induced samples. Statistical significance of the difference in proliferation rates between Dox induced samples was estimated using a two-tailed Welch’s t-test. Statistical significance of the luciferase assay was estimated by comparing the Dox induced sample to the un-induced sample using a two-tailed Welch’s t-test. To analyze the expression of apoptosis associated genes in HRA cells, we used BAD, BAK1, BAX, BBC3, BCL2, BCL2A1, BCL2L1, BID, BCL2L11, MCL1 and PMAIP1, all members of the Bcl2 family, BIRC2, BIRC3, BIRC5, BIRC6, BIRC7 and BIRC8 from the Inhibitor of Apoptosis family and CASP3, CASP6, CASP7, CASP9 and CASP10 among the caspases. Anova with 1000 permutations was used to estimate the significance of differential expression of each gene and a FDR cut-off of 0.05 was used to call significance. The analysis was performed using MEV and a bar plot depicting significantly differentially expressed genes among ND, 24+, 72+ and DW samples is shown in. Analysis of IHC staining was performed using STATA (www.stata.com/). The Wilcoxon Rank Sum Test was used to calculate the statistical significance of % Ki67 positive nuclei in the nNcL samples compared to the nNcH and nPcH samples. GenePattern *(* www.broadinstitute.org/cancer/software/genepattern/), GSEA *(* www.broadinstitute.org/gsea/) and MEV (www.tm4.org/) was used to analyze HRA gene expression data. Normalized gene expression data for the TCGA breast cancer samples was obtained from Firehose hosted at the Broad Institute, Cambridge, MA (http://gdac.broadinstitute.org/). Clinical data for the TCGA samples was obtained from the Cancer Genome Browser (https://genome-cancer.ucsc.edu/). Tumor purity data for the TCGA samples in the gene expression dataset was obtained from ESTIMATE (http://bioinformatics.mdanderson.org/main/ESTIMATE:Overview). Statistical analysis of the data was performed using STATA. A p-value < 0.05 was considered significant.

## Results

### Increasing RelA levels arrests proliferation of human mammary and fallopian tube epithelial cells

**Fig 1:**
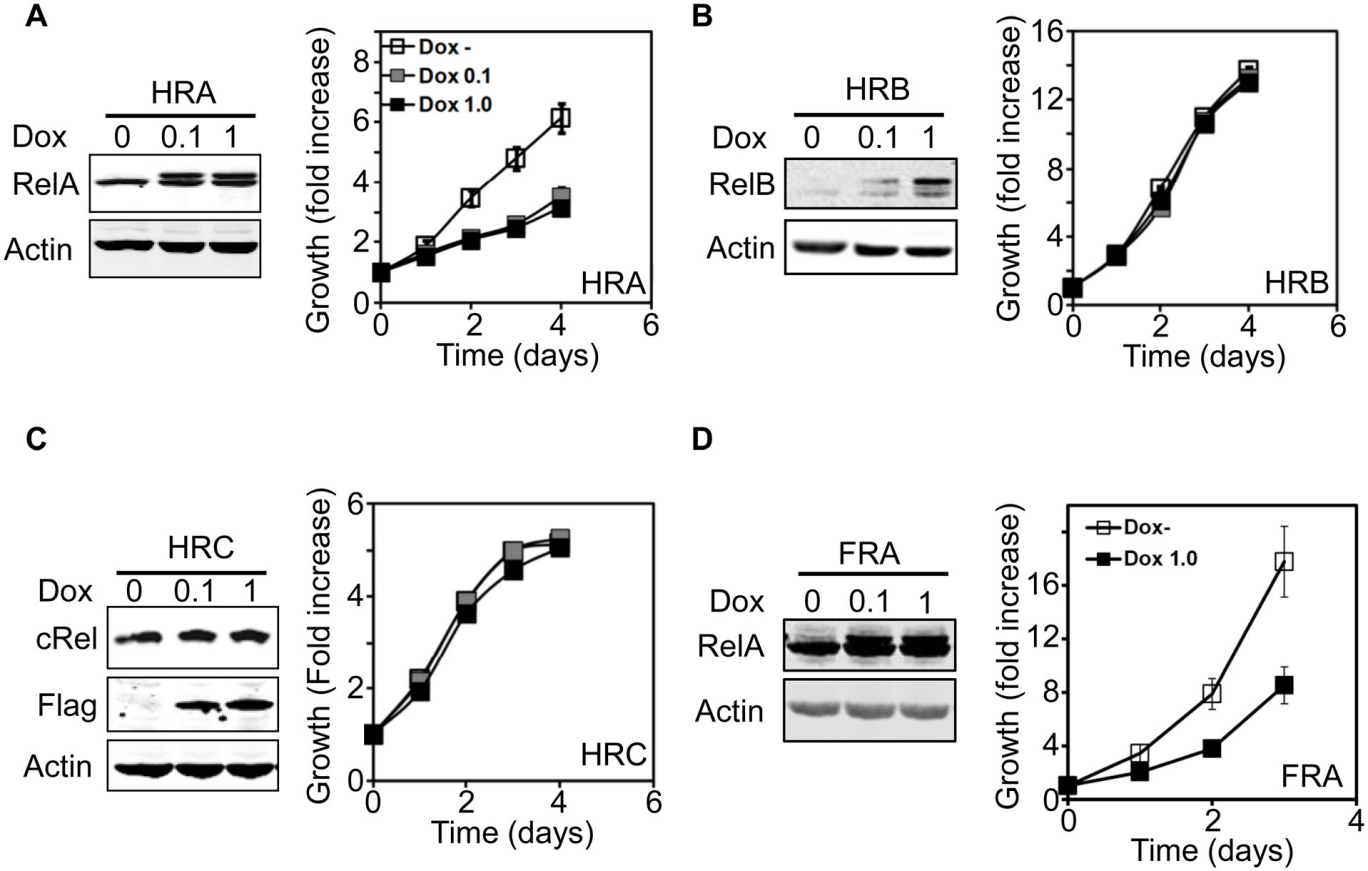
Conditional expression of RelA causes proliferation arrest in epithelial cells. **A—C**. Stable HMEC (A-C) conditionally expressing Flag-tagged RelA (HRA, A), RelB (HRB, B) or cRel (HRC, C) were generated and expression of the proteins in the presence of Dox (0.1 or 1 μg/ml) was verified by immunoblot. Flag-tagged RelA and RelB runs as a slower migrating band and induction of cRel was verified using an anti-Flag antibody. The effect of expressing RelA, RelB and cRel (compare Dox- with Dox 0.1/1.0 μg/ml) on HMEC proliferation was analyzed using the MTS assay. Absorbance values at each time was normalized to that obtained at the beginning of the experiment (Day 0) and plotted as fold increase over time. **D.** Similar to A except that RelA was expressed in Fallopian tube epithelial cells (FRA).

To evaluate the role of NF-kB in human epithelial cells, we used late-passage p16^neg^ hTERT immortalized human mammary epithelial cells, hitherto referred to as HMEC. HMEC were engineered to conditionally express Flag-tagged *RelA* under the Doxycycline- (Dox) inducible promoter (**H**MEC-**R**el**A**; HRA cells). After treatment with 0.1 and 1.0μg/ml of Dox, a higher molecular weight tagged RelA protein was detected in the HRA cells. HRA cells grew rapidly in the absence of Dox, but after treatment with either 0.1 or 1.0μg/ml of Dox, proliferation slowed dramatically (Fig 1A). Immunofluorescence staining showed majority of induced RelA was cytoplasmic (data not shown). We generated derivatives of HMECs that conditionally express Flag-*RelB* (**H**MEC-**R**el**B**, HRB cells) or Flag-*Rel* (**H**MEC-**R**el **C**, HRC cells). Dox dependent expression of RelB and cRel was confirmed and expression of neither resulted in proliferation arrest (Fig 1B and 1C).

Recent data suggests high-grade serous ovarian cancer may arise from fallopian tube epithelial cells [45]. We tested the effect of conditionally expressing RelA in immortalized primary fallopian tube epithelial cells (FT282 cells expressing RelA; FRA cells) [26]. Similar to HRA cells, Dox treatment of FRA cells induced RelA and inhibited proliferation (Fig 1D). We found no evidence for cleavage of PARP after induction of RelA in HRA cells for up to 60 hours, suggesting that the observed inhibition of proliferation is not due to apoptosis (S2A Fig). Since HMEC are grown in defined medium, we confirmed that RelA expression does not render the cells insensitive to growth factors in the medium by analyzing growth factor induced activation of ERK and AKT pathways. Induction of ERK and AKT activation were identical irrespective of the presence of RelA (S2B Fig). SV40-smallT antigen inhibits phosphatases, enhances growth factor receptor signaling and drives proliferation [46]. To test if SV40-smallT antigen could rescue the anti-proliferative effect of RelA, we generated a HRA derivative stably expressing SV40-smallT antigen (HRA-st cells). While HRA-st cells proliferate faster than HRA cells in the absence of Dox, addition of Dox and expression of RelA inhibited proliferation of HRA-st cells to the same extent as HRA cells (S2C Fig).

Therefore, among NF-kB factors encoding trans-activating domains, only RelA negatively regulates proliferation of epithelial cells. RelA dependent inhibition of proliferation cannot be rescued by increasing growth factor receptor signaling.

### Proliferation control by RelA is independent of p53 but dependent on Rb

**Fig 2:**
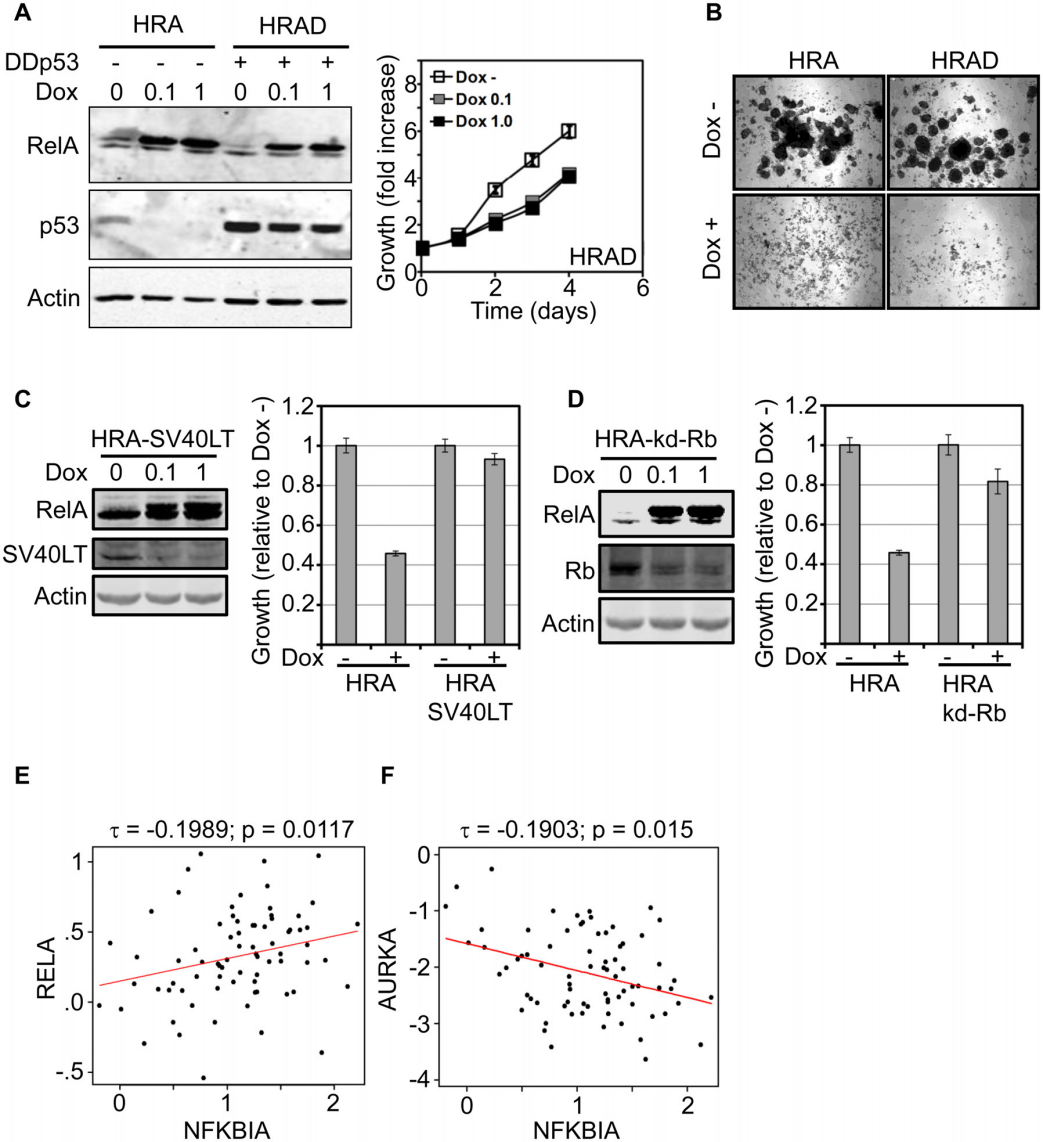
RelA induced proliferation arrest is p53 independent but Rb dependent. **A.** HRA cells constitutively expressing a short dominant negative form of p53 (DDp53, HRAD cells) was generated and expression of DDp53 was verified by the stabilization of endogenous p53. Proliferation assay was performed using HRAD cells in the presence or absence of Dox (0.1μg/ml or 1μg/ml) using the MTS assay. MTS values at each time-point was normalized to MTS values corresponding to initial amount of cells plated and plotted as fold increase in growth over time. **B.** HRA or HRAD cells were mixed with methocult medium supplemented with B27 and cultured in the absence and presence of Dox (1μg/ml) for 2 weeks. Representative images of colonies from a triplicate experiment are shown. **C.** HRA cells constitutively expressing SV40 Large T antigen (HRA-SV40LT) were generated and verified by immunoblot. HRA and HRA-SV40LT cells were plated in triplicates and cultured in the presence or absence of Dox (1μg/ml) for 3 days. Amount of cells under each condition was estimated using the MTS assay and normalized to values obtained in the un-induced samples. Rescue of RelA-induced proliferation arrest by SV40LT was statistically significant (p-value: 8.5 E -14). **D.** Double-conditional HRA cells expressing both RelA and a miR-shRNA targeting Rb (HRA-kd-Rb) were generated and verified by immunoblot. HRA and HRA-kd-Rb cells were plated in triplicates and proliferation of the cells was analyzed as in **C**. Rescue of RelA-induced proliferation arrest by knocking down Rb was statistically significant (p-value: 6.8 E -8). **E.** Scatter plot showing the correlation between expression of *RelA* and *NFKBIA* (IkB-α) in ER+/HER2- tumors with > 75% purity within the TCGA cohort. The Kendall-Tau correlation co-efficient and the associated p-values are shown. The linear regression of the correlation is indicated by the red line. **F.** Similar to the panel **E** except that the correlation is between AURKA, a marker for proliferation and NFKBIA.

The p53 and Rb pathways are major determinants of cell cycle control, response to cellular stress and are frequently disrupted in highly proliferating tumors. To test if proliferation arrest caused by RelA is dependent on p53 and/or Rb, we disrupted these pathways in HRA cells and analyzed the effect of RelA induction on proliferation. A dominant negative form (DDp53) encoding a short N-terminal fragment of p53 was stably introduced into HRA cells to disrupt p53 (HRAD cells) [47]. Dox induced expression of RelA was comparable in both HRA and HRAD cells and expression of DDp53 stabilized endogenous p53. However, inactivation of p53 did not rescue RelA dependent proliferation arrest (Fig 2A).

HMEC cultures contain progenitor/stem cells that form mammospheres under specific culture conditions [48]. We cultured HRA and HRAD cells under conditions that favor mammosphere formation and monitored the appearance of colonies. After two weeks in culture, multiple mammosphere-like colonies were observed in both HRA and HRAD cells in the absence of Dox. However, formation of mammosphere colonies was completely inhibited when RelA was induced in both HRA cells, and in HRAD cultures (Fig 2B).

Next, we destroyed both p53 and Rb pathways by generating a stable HRA derivative expressing SV40-large T antigen (HRA-SV40LT cells) and confirmed expression of both RelA and SV40LT by western blot (Fig 2C). Unlike HRA cells, induction of RelA in HRA-SV40LT cells did not result in proliferation arrest. To confirm that RelA induced proliferation arrest is RB dependent, we generated a double conditional HRA cell line harboring both inducible RelA and a miR-shRNA targeting Rb (HRA-kd-Rb). The sequence used to modify the pRXTN plasmid to conditionally express miR-shRNAs is given in (S3A Fig) [28]. Knock-down of Rb in HRA-kd-Rb cells rescued RelA induced proliferation arrest (Fig 2D). Therefore, RelA dependent proliferation arrest in normal epithelial cells is independent of p53 but dependent on competent Rb function.

Among breast tumors, ER+ tumors display a range of proliferation rates and grade while HER2+ and ER-/HER2- tumors proliferate faster and are usually high grade. Moreover, majority of ER+ tumors retain a functional Rb pathway. Therefore we tested the correlation between RelA levels and proliferation among ER+ tumors in the TCGA gene expression dataset. Since RelA is widely expressed, we first screened TCGA samples based on tumor purity derived using the ESTIMATE procedure [42]. Tumor purity in the TCGA samples ranges from 17% to 100% (S3B Fig). Using a tumor purity cut-off of 75% retained sufficient samples (75 samples) within the ER+/HER2- subtype for analysis. Aurora kinase A (*AURKA*) is a single gene predictor of proliferation within the ER+ subtype of breast cancer [49]. The Kendall-Tau correlation between AURKA and RelA in ER+/HER2- tumors was negative and insignificant (S3C Fig). Since RelA mRNA levels may not correlate with protein levels, we used mRNA levels of IkBα (*NFKBIA*), a direct target of RelA, as a surrogate for *RelA* expression and activity. The Kendall-Tau correlation between *RelA* and *NFKBIA* mRNA levels was significant and positive (Fig 2E). The correlation between *AURKA* and *NFKBIA* was also significant but negative, confirming the inclination of RelA to restrain proliferation (Fig 2F).

### RelA induction down-regulates CDK4 resulting in Rb hypo-phosphorylation and cell cycle arrest

**Fig 3:**
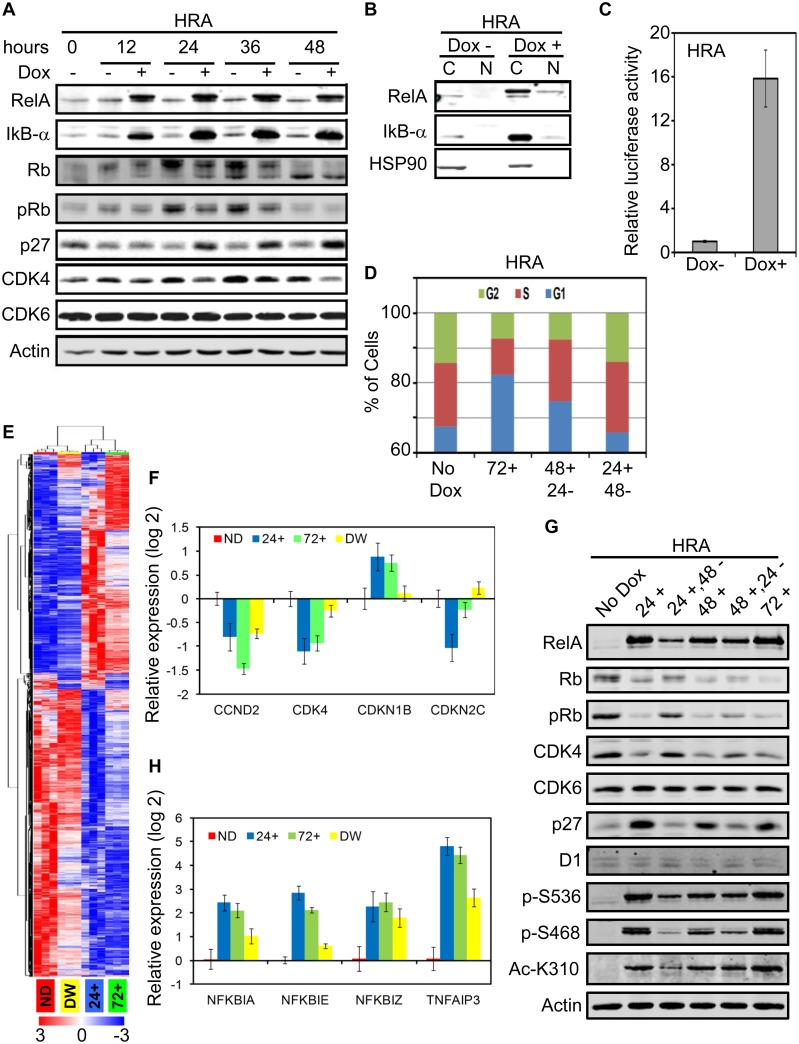
RelA induction down-regulates CDK4 resulting in Rb hypo-phosphorylation and cell cycle arrest. **A.** HRA cells were cultured in the presence and absence of Dox (1μg/ml) for 48 hours and changes in indicated proteins was monitored every 12 hours by immunoblot. **B.** HRA cells were cultured in the presence and absence of Dox (1μg/ml) for 48 hours and cytoplasmic (C) and nuclear (N) proteins were extracted. The samples were analyzed by immunoblot. HSP90 was used as a marker for purity of the nuclear fraction. **C.** HRA cells were co-transfected with an NF-kB reporter Firefly luciferase and background control Renilla luciferase plasmids. Transfected cells were trypnized, plated in triplicates and cultured in the presence or absence of Dox (1μg/ml) for 48 hours. Subsequently, expression of Firefly and Renilla luciferase was estimated. Activity of Firefly luciferase in the Dox+ and Dox– samples were normalized to the respective activity of Renilla luciferase. Activity values in the Dox+ sample was normalized to the Dox- sample to obtain the bar plot. Increase in Firefly Luciferase activity following RelA induction was statistically significant (p-value: 1.3 E -7). **D.** HRA cells were cultured in the absence (No Dox) or presence of Dox (1μg/ml) for 72 hours (72+). Parallel HRA cells cultured in the presence of Dox for 48 or 24 hours and then switched to medium devoid of Dox for 24 (48+, 24-) or 48 (24+, 48-) hours were fixed and stained using Propidum Iodite. Distribution of cells in the G2, S and G1 phases of the cell cycle was analyzed using flow cytometry. **E.** HRA cells were treated according to the scheme in S4A Fig. Total RNA was extracted from the samples and global gene expression levels was determined using the Affymetrix Primeview array. Differentially expressed genes among the four conditions were identified (FDR < 0.05) and hierarchical clustering of the genes and samples was performed using the differentially expressed genes. **F.** Expression levels of the core G1/S transition genes including cyclins, CDKs and CDK inhibitors in 24+, 72+ and DW samples were normalized to the expression level in the ND samples. The bar plot indicates differentially expressed genes (p-value <0.05, fold change > 2) from the core G1/S transition gene list. **G.** HRA cells were treated with dox according to the scheme in S4C Fig. Whole cell lysates were analyzed using indicated antibodies, including antibodies specific to phosphorylated (S536 and S468) and acetylated (K310) forms of RelA. **H**. Differential expression (p-value < 0.05, fold change > 4) of feedback negative regulators of RelA including IkB, CYLD and TNFAIP3 were identified. The bar plot shows expression changes relative to the ND sample.

Phosphorylation of Rb triggers cell-cycle progression through G1 and into S-phase [50]. We analyzed Rb phosphorylation and levels of core G1/S transition genes in HRA cells after induction of RelA at 12 hour intervals up to 48 hours. RelA induction caused dramatic up-regulation of IkB-α, which is both an inhibitor and transcriptional target of RelA. Phosphorylation of Rb is diminished in the Dox induced samples compared to the un-induced samples. By 48 hours, cells grown in the absence of Dox become contact-inhibited and Rb becomes hypo-phosphorylated, as expected in cultures of primary cells. Together with Rb hypo-phosphorylation, Cyclin-dependent kinase (CDK) inhibitor 1B (*CDKN1B*, p27) level is elevated and CDK4 levels drop in RelA induced cells while CDK6 levels do not change (Fig 3A). Proliferation arrest caused by RelA expression is due to Rb hypo-phosphorylation and activation of the G1/S checkpoint. Diminished levels of CDK4 and increase in p27 may be responsible for Rb hypo-phosphorylaion.

Expression of RelA in pro-B cells induces proliferation arrest and requires both DNA binding and trans-activating domains of RelA for this effect [18]. To test if expressed RelA is transcriptionally active, we analyzed nuclear translocation of RelA after induction. IkB-α is strongly induced by RelA, and found principally in the cytoplasm as expected. While, the majority of expressed RelA was cytoplasmic, a minor fraction was detected in the nucleus. HSP90 is found exclusively in the cytoplasmic fraction and confirms the purity of the nuclear fraction (Fig 3B). This minor fraction of RelA was sufficient to induce more than a 15-fold increase in luciferase expression driven by an NF-kB promoter (Fig 3C). These results confirm that a fraction of the induced RelA translocates to the nucleus and is transcriptionally active.

One advantage of an inducible system is reversibility. The anti-proliferative effect of RelA is already evident at 24 hours post induction and increase in cell number at later time points in the continued presence of Dox is minimal (Fig 1A). Therefore, we tested reversibility of the proliferation arrest induced upon RelA induction by flow cytometry by withdrawing Dox after 24 and 48 hours of induction. A system was devised to harvest cells under conditions where media changes are identical in both control and Dox-induced cultures. In HRA cells cultured in the absence of Dox for 72 hours (No Dox), 68% of the cells were in G0/G1 and this fraction increased to 82% when the cells were cultured in the presence of Dox for 72 hours (72+). Withdrawal of Dox for 24 hours after growing the cells in the presence of Dox for 48 hours (48+, 24-), decreased the G0/G1 fraction to 75%. Withdrawal of Dox for 48 hours post induction for 24 hours (24+, 48-), the cultures reverted to a cell-cycle distribution similar to the un-induced sample (24+, 48-). (Fig 3D). These results indicate that changes in the RelA induced transcriptome is reversed within 48 hours of Dox withdrawal.

To identify the molecular determinants of RelA dependent cell cycle arrest, we analyzed changes in global gene expression profile of HRA cells cultured in the absence, presence and after withdrawal of Dox. Triplicate samples for gene expression analysis were generated according to the scheme in S4A Fig. HRA cells were plated and 24 hours later all cultures underwent medium change with or without Dox. Black arrows indicate addition of Dox, green arrow indicates withdrawal of Dox, red arrows indicate times when the cultures were harvested for RNA extraction and filled bars indicate period of Dox treatment. The time points in the experiment are shown at the bottom. Triplicate samples with no Dox (ND) treatment, treated with Dox for 24 hours (24+) or 72 hours (72+) and treated with Dox for 24 hours and maintained in the absence of Dox for a further 48 hours (Dox Withdrawn; DW) were generated. The gene expression data was analyzed using TMEV and GenePattern as described in the experimental methods section. LIMMA identified 3030 genes as differentially expressed among the four groups with FDR q-value < 0.001. Hierarchical clustering of the differentially expressed genes divided the samples into two major groups. The ND and DW samples clustered together, as did the 24+ and 72+ samples (Fig 3E).

After induction of RelA for either 24 or 72 hours, changes in gene expression profile of the cells was dramatic. Withdrawal of Dox for 48 hours after induction for 24 hours (DW samples) caused the vast majority of differentially expressed genes to revert to ND levels; the expression of only 214 genes remained significantly different in the DW samples compared to ND (S4B Fig). These findings corroborate restoration of cell-cycle progression after Dox withdrawal and suggests that RelA induction does not provoke a permanent program of altered gene expression, such as in cell senescence.

Since the anti-proliferative effect of RelA is Rb dependent and correlates with Rb phosphorylation status, we analyzed expression changes in the core G1/S transition genes: cyclins, CDKs and CDK inhibitors. Among these genes, only *CCND2* (cyclin D2), *CDK4*, *CDKN1B* (p27) and *CDKN2C* (p18/INK4c) were differentially expressed (p-val < 0.05, fold change > 2) (Fig 3F). The reversal in expression levels of *CDK4* and *CDKN1B* in the DW samples was striking and may be responsible for reversal of proliferation arrest upon Dox withdrawal.

If down-regulation of CDK4 and up-regulation of p27 is responsible for cell cycle arrest, Dox withdrawal, which restores cell-cycle distribution, should also restore CDK4 and p27 to levels seen in un-induced cells. We used a Dox treatment and withdrawal scheme, which minimizes the effect of medium change, to monitor changes in Rb phosphorylation, and CDK4 and p27 levels (S4C Fig). Within 24 hours of Dox induction, CDK4 levels drop, p27 levels increase and Rb becomes hypo-phosphorylated. Levels of these proteins do not change during 72 hours of Dox exposure. Dox withdrawal progressively re-establishes Rb phosphorylation, restores CDK4, restrains p27, but does not affect CDK6 (Fig 3G). These changes parallel the restoration of G0/G1, S and G2/M fractions seen in Fig 3D and the reversal of global gene expression pattern seen in Fig 3E upon Dox withdrawal. CyclinD1 levels do not change and is unlikely to be the driver of changes in proliferation.

Post-translational modification of RelA are required for transcriptional activity [51]. Therefore, we analyzed phosphorylation changes in IkB-α, IKK and RelA and the expression changes in feedback negative regulators of RelA target genes. Although IkB-α is strongly induced by RelA, we did not detect the phosphorylated form of the protein. Furthermore, we did not detect phosphorylation and activation of the IKKs either (data not shown). Induced RelA was phosphorylated at S536, S468 and acetylated at K310, all markers for transcriptional activation [51]. Dox withdrawal for 48 hours after induction for 24 hours caused marked reduction in levels of phosphorylation at S468 and acetylation at K310 consistent with reversal of target gene expression observed in the DW sample (Fig 3G).

**Fig 4:**
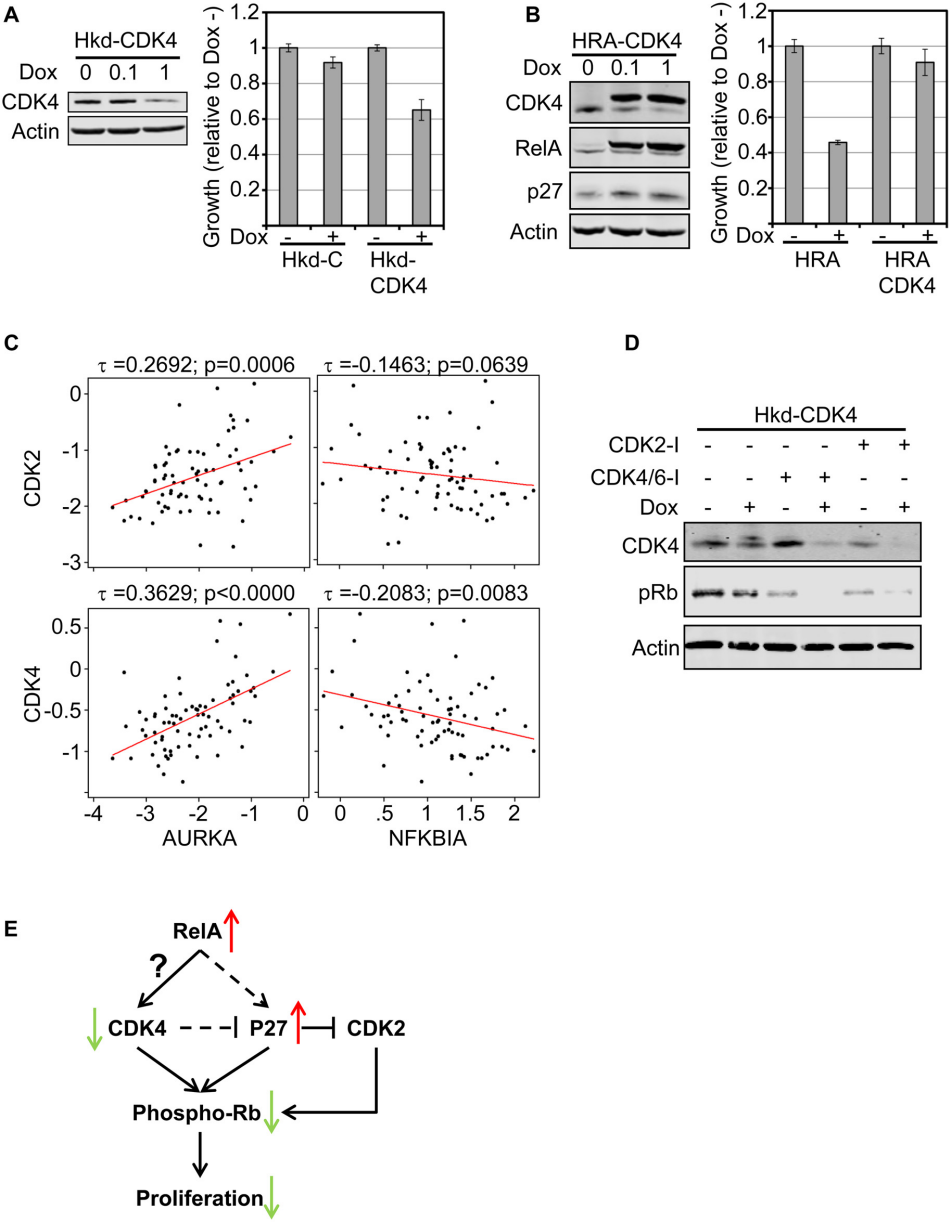
Restoring CDK4 rescues RelA induced proliferation arrest. **A.** Stable HMEC cells harboring the Tetracycline promoter trans-activator expressing a miR-shRNA targeting CDK4 (Hkd-CDK4) or a control miR-shRNA (Hkd-C) were generated. Immunoblot confirmed knock-down of CDK4 after treatment with Dox (1μg/ml) for 48 hours. Proliferation of Hkd-C and Hkd-CDK4 was measured after inducing expression of both miR-shRNAs for 72 hours using the MTS assay. The MTS values were normalized to the Dox- samples for each cell line. Standard error of a triplicate experiment is shown. Dox dependent decrease in proliferation of Hkd-CDK4 was statistically significant compared to Hkd-C cells (p-value: 0.001). **B.** Double conditional HRA cells expressing the CDK4 ORF was generated (HRA-CDK4) and induction of both proteins was verified by immunoblot. Proliferation assay was performed with HRA and HRA-CDK4 cells as described in **A**. Rescue of RelA-induced proliferation arrest by co-expressing CDK4 was statistically significant (p-value: 4.8 E -8). **C.** Correlation in expression between the indicated genes was estimated using Kendall-Tau in ER+/HER2- tumors analyzed by TCGA. The Tau correlation co-efficient and associated p-values are shown. The linear regression line (red) is also indicated. **D.** Hkd-CDK4 cells were plated and 12 hours later, expression of miR-shCDK4 was induced for 24 hours using Dox (1μg/ml) as indicated. Following incubation with Dox, the cells were treated with PD0332991 (CDK4/6 inhibitor, 200nM) or CVT-313 (CDK2 inhibitor, 5μM) as indicated for 12 hours. After which, whole cell lysates were prepared and changes in CDK4 and phospho-Rb was analyzed by immunoblot. **E.** Schematic diagram describing the results so far. When RelA levels are increased, CDK4 levels drop, p27 levels increase. Increase in p27 suppresses CDK2 activity. Diminished levels of CDK4 and inhibition of CDK2 by p27 results in decrease in phosphor-Rb and consequently proliferation. CDK4 may regulate p27 levels.

Upon RelA induction, negative feedback regulators *NFKBIA* (IkB-α), *NFKBIE* (IkB-ε), *NFKBIZ* and *TNFAIP3* (A20) were differentially expressed (p-val <0.05, fold change > 4) while expression changes in other IkBs, Bcl3 and CYLD were below this threshold (Fig 4H). Except NFKBIE, expression of the other negative regulators continue to be greater than two-fold in the DW samples compared to ND samples. Robust and sustained induction of TNFAIP3 may be responsible for the lack of activation of the IKK-signalosome, while, elevated IkB-α may rapidly bind and sequester RelA in the cytoplasm once Dox is withdrawn [2]. While the identity of the kinases that phosphorylate RelA under these conditions is not known, the data are consistent with increased basal transcriptional activity of RelA when RelA levels are high.

In conclusion, increasing RelA expression levels results in higher basal transcriptional activity, alters expression of over 3000 genes and incites a block in G1/S transition. The proliferation arrest induced by RelA is most likely a consequence of CDK4 down-regulation and up-regulation of p27 which results in Rb hypo-phosphorylation. When RelA expression is terminated by Dox withdrawal, negative feedback inhibitors induced by RelA rapidly suppresses RelA transcriptional activity, majority of the genes induced by RelA revert to pre-induction levels and the cells resume normal growth. A similar mechanism may be operating in ER+/HER2- breast tumors that express high levels of RelA resulting in diminished proliferation.

### Restoring CDK4 rescues RelA induced proliferation arrest

Coordinated activity of CDK2, CDK4 and CDK6 phosphorylate Rb and drive cells across the G1/S checkpoint. Among the CDKs, the CyclinD1/CDK4 activity is essential for mammary development and HER2neu induced tumors [52, 53]. Among the CDKs, only CDK4 levels drop upon RelA induction while up-regulation of p27 may inhibit CDK2 [54]. To determine if HMEC are dependent on CDK4, we depleted CDK4 using a miR-shRNA targeting CDK4 (Hkd-CDK4 cells) or a control miR-shRNA (Hkd-C cells; Fig 4A).

Upon Dox treatment, CDK4 levels dropped in Hkd-CDK4 cells and resulted in reduced proliferation in comparison to Hkd-C cells treated with Dox. Providing an excess of CDK4 may rescue RelA induced proliferation arrest. We generated double conditional cells where Dox induction results in expression of both RelA and CDK4 (HRA-CDK4 cells) and expression of both upon induction was confirmed (Fig 4B). In HRA-CDK4 cells, RelA induction reduced expression of endogenous CDK4, but the exogenous CDK4 rescued the RelA induced proliferation arrest. Furthermore, co-expression of CDK4 and RelA did not result in increased p27 levels seen after inducing RelA, implicating CDK4 in regulating p27 protein levels after introduction of RelA.

We speculated that lower proliferation rates in ER+/HER2- breast tumors with high levels of RelA may be due to diminished CDK4 levels. We analyzed the correlation of *CDK2* and *CDK4* with *AURKA*, a surrogate for proliferative fraction, in the TCGA cohort of breast tumors with an estimated tumor purity of >75% (like in Fig 2E and 2F). Expression of *CDK2* and *CDK4* correlates with *AURKA*, was positive and highly significant. The correlation between CDK6 and AURKA in this cohort was insignificant (data not shown). CDK4 may be the prominent driver of proliferation in the ER+/HER2- subtype of breast tumors. We used *NFKBIA* (IkB-α) expression as a surrogate for basal RelA activity in this cohort of ER+/HER2- tumors. The correlation between *NFKBIA* and *CDK4* was the most significant among the three CDKs and was negative (Fig 4C and data not shown). These findings in the TCGA dataset supports our observation in HRA cells.

CDK4/6 inhibitors have shown favorable activity in combination with letrozole for treating patients with advanced ER+ breast tumors [55]. Biomarkers of response to the inhibitors are not known, other than strict requirement for an active Rb pathway [56]. We analyzed Rb phosphorylation in the presence and absence of the CDK4/6 inhibitor Palbociclib, the CDK2 inhibitor CVT-313 on Hkd-CDK4 cells in the presence and absence of Dox. Knocking down CDK4 augmented the effect of CDK4/6 and CDK2 inhibitors on Rb phosphorylation (Fig 4D).

Expression of RelA in HMEC diminishes CDK4 levels and causes cell cycle arrest in G1. Elevation in the level of p27 observed after RelA induction may itself be a consequence of diminished CDK4. Together, reduced CDK4 and elevated p27 cause hypo-phosphorylation of Rb and arrest cell-cycle progression in G1. CDK4 levels in ER+/HER2- breast tumors may similarly be negatively regulated by RelA. RelA down-regulation of CDK4 expression may restrain proliferation, and perhaps signal higher response to CDK4 inhibitors in patients with ER+/HER2- tumors. Left unexplained is the way in which RelA influences the expression of CDK4 (Fig 4E).

### RelA induced interferon response may be responsible for CDK4 down-regulation and proliferation arrest

**Fig 5:**
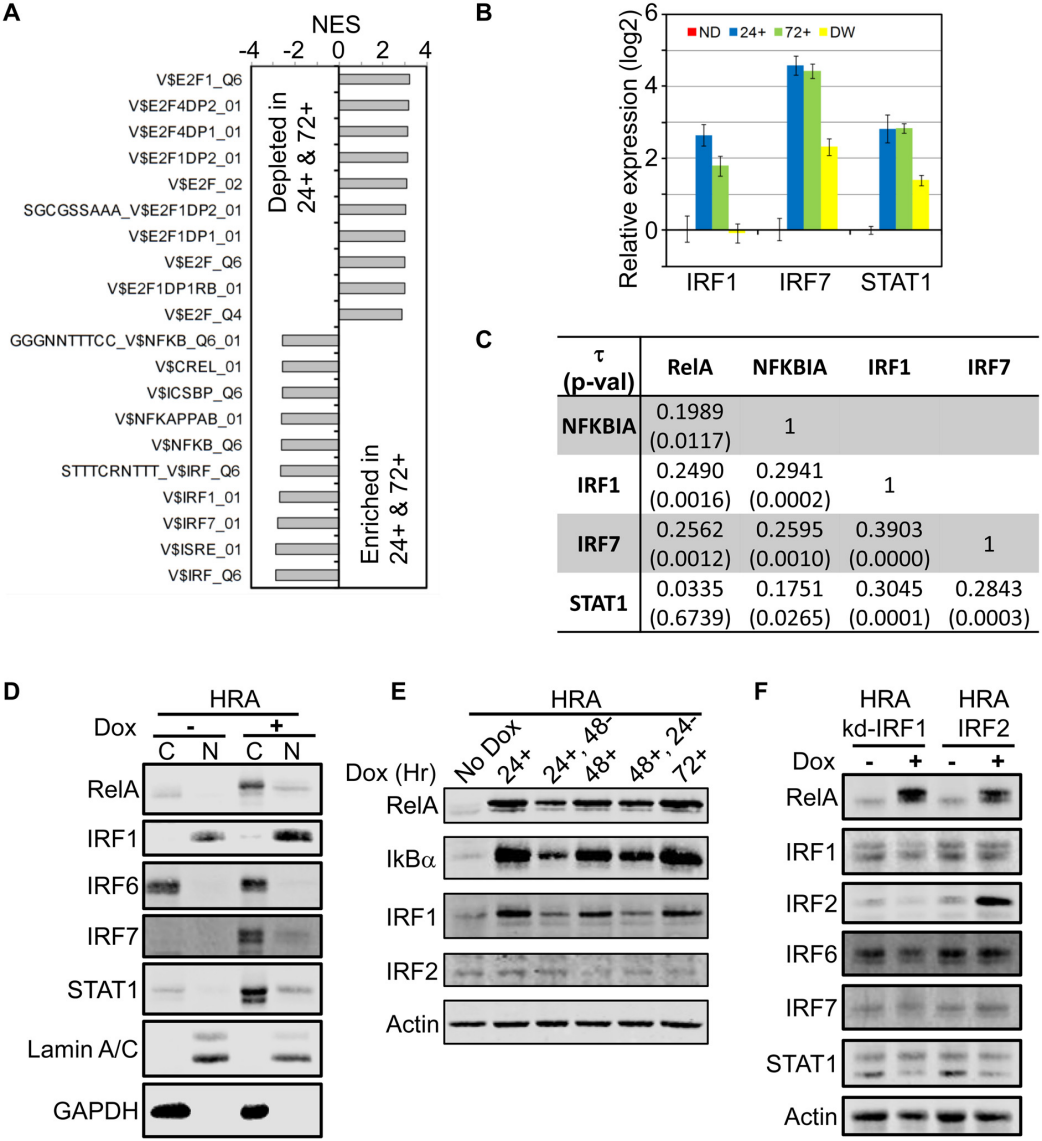
RelA induced interferon response may be responsible for CDK4 down-regulation and proliferation arrest. **A.** Samples used to generate gene expression data were divided into two groups (ND/DW, and 24+/72+) and Gene Set Enrichment Analysis (GSEA) was performed on the differentially expressed genes using the motifs gene-set database from MsigDB. Top 10 gene sets (FDR q-value = 0) and their associated normalized enrichment scores (NES) are shown. **B.** Differentially expressed transcription factors (p-value <0.05, fold change > 4) that could regulate motifs identified in **A** are shown. Expression values were normalized to the ND sample. **C.** The Kendall-Tau correlation and associated p-value between indicated genes in the TCGA cohort of ER+/HER2- tumors (> 75% purity). **D.** Cytoplasmic (C) and nuclear (N) extracts of HRA cells cultured in the presence and absence of Dox for 48 hours was analyzed by immunoblot using indicated antibodies. **E.** HRA cells were cultured according to the scheme in S4C Fig and whole cell lysates were analyzed by immunoblot using indicated antibodies. **F.** Stable double conditional HRA cells expressing RelA and either a miR-shRNA targeting IRF1 (HRA-kd-IRF1 cells) or the IRF2 ORF (HRA-IRF2 cells) were generated. Expression of miR-shIRF1 or IRF2 was induced for 48 hours following which whole cell extracts were prepared and analyzed by immunoblot using indicated antibodies.

Despite its regulation by RelA, CDK4 is not a known target of RelA. We hypothesized an intermediate transcription factor induced by RelA regulates CDK4 expression and used Gene Set Enrichment Analysis (GSEA) to identify candidates [39]. We combined No Dox (ND) and Dox withdrawal (DW) samples, and the Dox treatment replicates at 24+ and 72+ hours of Dox exposure to generate two groups for comparison. Multiple E2F factor binding motifs were identified in genes down-regulated in 24+ and 72+ samples, while motifs for the Interferon Response Factors (IRF) and NF-kB factors were up-regulated (Fig 5A).

Down-regulation of E2F target genes in 24+ and 72+ samples is consistent with RelA-dependent cell-cycle arrest. Up-regulation of NF-kB target genes in 24+ and 72+ samples is expected. However, enrichment for interferon target genes was unexpected. Among the factors that could potentially regulate the identified interferon regulated motifs, differential expression of IRF1, IRF7 and STAT1 was significant (p-value <0.05; fold change > 4). Induction of IRF1 mRNA follows kinetics of basal transcriptional activity of RelA, with a dramatic return to unexpressed levels after Dox withdrawal. IRF7 and STAT1 mRNA levels do not drop to ND levels upon Dox withdrawal (Fig 5B). Therefore, up-regulation of interferon target genes upon RelA induction may result from the coordinated activities of IRF1, IRF7 and STAT1.

If the RelA-IRF transcriptional network is extant and regulates CDK4 expression, ER+/HER2- tumors that exhibit a range of proliferation rates should retain this network. We examined ER+/HER2- tumors with > 75% purity in TCGA, and determined the Kendall-Tau correlation co-efficient for expression of *RelA*, *NFKBIA*, *IRF1*, *IRF7* and *STAT1*. While *RelA* mRNA levels correlated with *NFKBIA*, *IRF1* and *IRF7*, the correlation between *NFKBIA* (IkB-α) and *IRF1*, *IRF7* and *STAT1* was higher. There was high correlation between expression of the three interferon factors themselves, suggesting cross-regulation (Fig 5C).

Over-expression of IRF1, IRF5, IRF6 and IRF7 cause cell cycle arrest in various cell types [57–59]. IRF7 and STAT1 are cytoplasmic proteins that translocate to the nucleus following activation of the interferon pathway, while IRF1 is predominantly nuclear [60]. We analyzed nuclear and cytoplasmic localization of interferon transcription factors in protein extracts from HRA cells induced with Dox for 48 hours. RelA induction resulted in robust expression of IRF1, IRF7 and STAT1 while there was no change in the level of IRF6, consistent with their pattern of gene expression. Among these factors, almost all of the basal and induced IRF1 was present in the nucleus while IRF6 was predominantly cytoplasmic. The majority of IRF7 and STAT1 were cytoplasmic, although small amounts of higher migrating forms of both proteins were detected in the nuclear fraction (Fig 5D).

The interferon pathway is activated after ligands engage receptors leading to expression and activation of cognate transcription factors. All of the type I-III interferon receptors are expressed at appreciable levels in HMEC cells and available for activation. Among the interferon ligands, only *IFNL1* (IFNλ-1) is expressed robustly in HMEC after induction of RelA and its expression follows RelA induction kinetics (S5A Fig). Although it is possible IFNλ-1 is activating the interferon response, RelA induction results in modest nuclear translocation of IRF7 and STAT1 (Fig 5D). Since almost all of the induced IRF1 is nuclear, IRF1 may be the dominant factor mediating the interferon response observed after RelA induction.

*IRF1* is a *bona fide* target of RelA and has multiple NF-kB binding motifs in its promoter sequence (S5B Fig) [61]. Over-expression of IRF1 inhibits proliferation by suppressing the expression of CDK2, CDK4 and up-regulating the cell cycle inhibitor p21 [62, 63]. Since RelA induced proliferation arrest is reversible, and *IRF1* mRNA levels highly correlate with basal transcriptional activity of RelA, we tested if IRF1 protein levels correlates with its mRNA levels. HRA cells were treated with Dox according to the scheme in S4C Fig. Induction of RelA in HRA cells increased IRF1 protein at 24, 48 and 72 hours. Levels of IRF1 declined to near un-induced levels after withdrawal of Dox for 48 hours (24+, 48-), consistent with its mRNA profile and short half-life (Fig 5E; [64]). IRF2 competes with IRF1 at interferon consensus sequences and inhibits IRF1 transcriptional activity [65]. IRF2 protein levels do not change with RelA induction and withdrawal. Down-regulation of IRF1 in 24+, 48- sample to un-induced (No dox) levels further supports decrease in basal transcriptional activity of RelA after Dox withdrawal.

To determine the hierarchy of transcriptional induction of IRF1, IRF7 and STAT1 post RelA induction and how IRF2 may alter this network, we generated stable double conditional HRA lines expressing a miR-shRNA targeting *IRF1* (HRA-kd-IRF1) or expressing *IRF2* (HRA-IRF2 cells). In HRA-kd-IRF1 cells, 48 hours of Dox treatment induced RelA but prevented IRF1 induction. Preventing the induction of IRF1 suppressed expression of IRF7 and STAT1. In HRA-IRF2 cells, both RelA and IRF2 were induced by Dox, while IRF1, IRF7 and STAT1 was not induced (Fig 5F). Changes in the level of IRF6 was minor. This result suggests that RelA directly induces IRF1, and expression of IRF7 and STAT1 are downstream of IRF1. Furthermore, IRF2 seems to alter the ability of RelA to induce IRF1, since IRF1 levels remain low after RelA induction in the double conditional HRA-IRF2 cells.

IRF1 is a crucial mediator of Tamoxifen-induced apoptosis of HPV-16 E6 immortalized HMEC [66]. IRF1 mediated activation of caspase 1 and 3 was responsible for induction of apoptosis in these cells [63]. Similarly, apoptosis induced by Tamoxifen in breast cancer cell lines is dependent on IRF1 [67]. Expression of IRF1 in murine breast tumor cells promotes apoptosis and inhibits tumor growth [68]. Induction of RelA in HRA cells for up to 60 hours showed no evidence of apoptosis like membrane blebbing or floating cells, and there was no evidence of PARP cleavage. To further rule out apoptosis in Dox induced HRA cells, we analyzed gene expression patterns of prominent pro and anti-apoptotic proteins. The gene list included Bcl2 family members, IAPs and caspases and the analysis was performed as described in materials and methods [69–71]. Induction of BCL2A1, an anti-apoptotic gene and a target of RelA was dramatic (> 200 fold) in the 24+ and 72+ samples. BCL2A1 binds and inactivates multiple pro-apoptotic Bcl2 family proteins including BIM, PUMA, BID, NOXA, BAX and BAK [69]. BIRC3 (cIAP), another anti-apoptotic gene was upregulated > 10 fold in the Dox treated samples (S4D Fig) [72]. Expression changes in other members of the BCL2 family proteins, caspases and IAPs was modest. Robust anti-apoptosis induced by RelA may overcome the pro-apoptotic activity of IRF1 and maintain cells in a quiescent state.

In conclusion, induction of RelA in HMEC results in potent expression of IRF1, and IRF1 up-regulates IRF7 and STAT1. In turn, CDK4 levels fall, Rb becomes hypo-phosphorylated, the Rb checkpoint is activated, E2F target genes are down-regulated and cells arrest in G1. Withdrawal of Dox reduces basal transcriptional activity of RelA, restrains IRF1 expression, and the interferon response downstream of IRF1 is terminated and the cells return to cycling. Interferons are known modulators of the cell cycle and engaging the interferon pathway by RelA is likely responsible for down-regulating CDK4 [65]. IRF1 integrates RelA and interferon signaling pathways.

### RelA and IFN-γ converge on IRF1 to suppress CDK4 and inhibit proliferation

**Fig 6:**
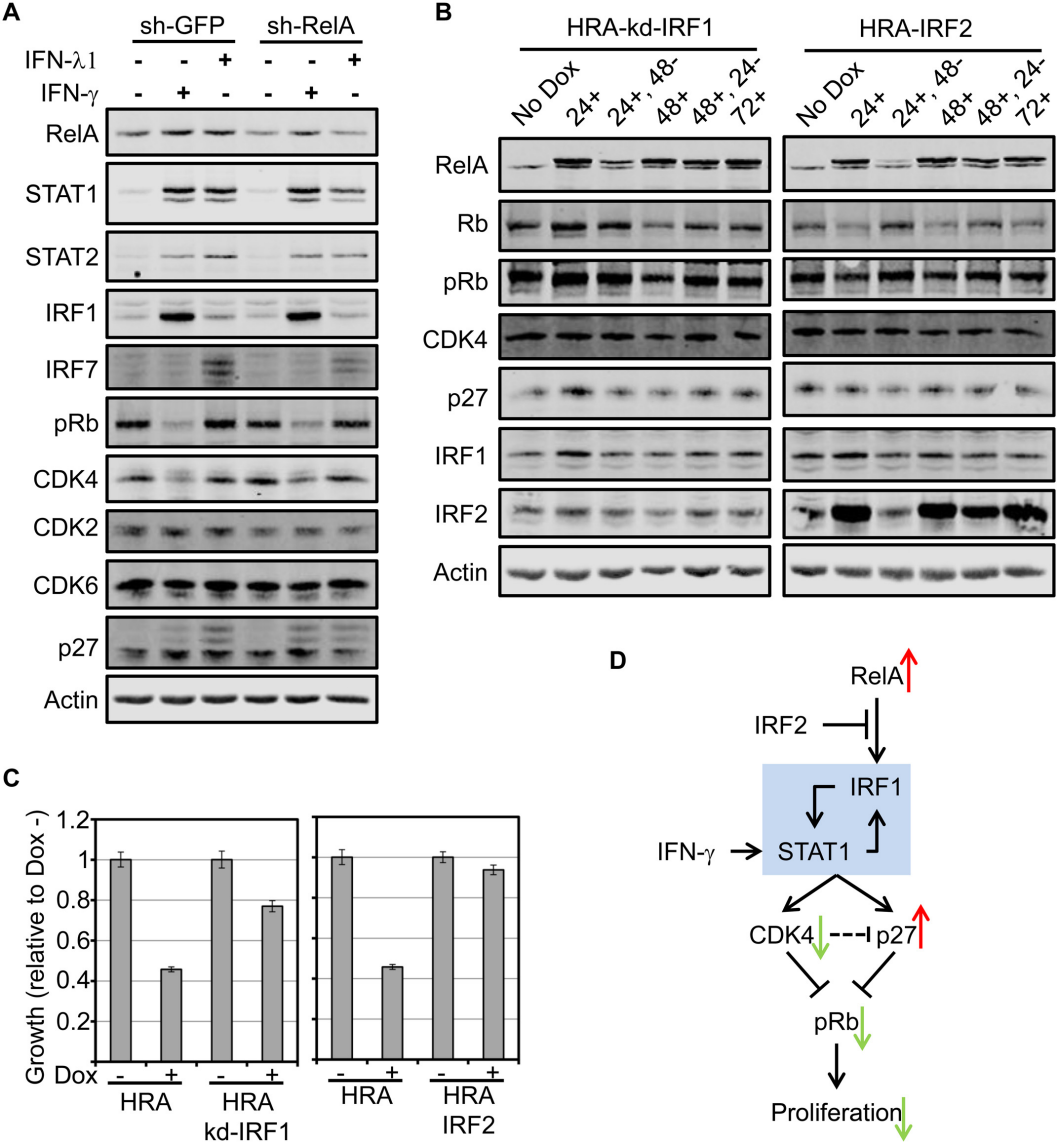
RelA and IFN-γ converge on IRF1 to suppress CDK4 and inhibit proliferation. **A.** Stable HMEC expressing sh-GFP or sh-RelA were generated. The cells were treated with IFN-λ1 of IFN-γ for 48 hours following which whole cell extracts were prepared and analyzed by immunoblot using indicated antibodies. **B.** HRA-kd-IRF1 and HRA-IRF2 cells were cultured according to the scheme in S5C Fig and whole cell lysates were analyzed by immunoblot using indicated antibodies. **C.** Proliferation assay was performed using HRA, HRA-kd-IRF1 and HRA-IRF2 cells in the presence and absence of Dox as indicated. The bar plots indicate growth of cells in the span of 72 hours normalized to growth in the absence of Dox. Error bars indicate triplicate samples. Rescue of RelA-induced proliferation arrest by knocking down IRF1 or co-expressing IRF2 was statistically significant (p-value: 3.4 E -12 and 1.1 E -15 respectively). **D.** Schematic representation of the findings in this study. Increased basal activity of RelA when expressed at high levels in epithelial cells and tumors induces IRF1. Increase in IRF1 causes up-regulation of Interferon Response Factors like STAT1 and orchestrates an interferon response. The interferon response down-regulates CDK4 and up-regulates of p27 leading to Rb hypo-phosphorylation which suppresses proliferation. Stimulation with IFN-γ elicits a similar response except that IRF1 induction is directed through STAT1. The IRF1-STAT1 positive feedback circuit may be essential for the interferon response and suppression of proliferation.

Stimulation of HMEC with IFN-γ causes down-regulation of CDK4, upregulation of p27, hypo-phosphorylation of Rb and cell cycle arrest in G1 [73]. Expression of RelA in HMEC upregulates IFN-λ1 and has similar phenotypic and molecular effects on the cells. To test if IFN-λ1 stimulation of HMEC down-regulates CDK4 and if RelA is required for IFN-γ or IFN-λ1 to down-regulate CDK4, we stably knocked down RelA in HMEC (sh-RelA) and achieved RelA knock-down levels of about 40%. HMEC harboring a shRNA targeting GFP (sh-GFP) was used as the control. Stimulation of both lines with IFN-γ for 48 hours caused up-regulation of STAT1, STAT2 and IRF1 but not IRF7, irrespective of RelA levels. Furthermore, IFN-γ caused down-regulation of CDK4 and hypo-phosphorylation of Rb, independent of RelA levels (Fig 6A).

Stimulation of sh-GFP and sh-RelA cells with IFN-λ1 induced the expression of STAT1 STAT2 and IRF7 but not IRF1. Moreover, reduction in RelA levels diminished the induction of STAT1, STAT2 and IRF7. However, we observed no changes in CDK4 levels nor Rb phosphorylation after IFN-λ1 stimulation of both cell lines (Fig 6A).

RelA induction upregulates IRF1, IRF7 and STAT1, and down-regulates CDK4. IFN-γ stimulation of HMEC induces STAT1, STAT2 and IRF1 while IFN-λ1 stimulation induces STAT1, STAT2 and IRF7. Only IFN-γ stimulation down-regulated CDK4. Therefore CDK4 down-regulation is contingent upon IRF1 expression. IRF7 induction in HRA cells may be due to the activity of IFNλ1 either directly induced by IRF1 or through transcription factors induced by IRF1. These results suggest that RelA expression and IFN-γ stimulation independently converges on IRF1 to down-regulate CDK4 and arrest proliferation.

Based on these results, we postulated that knocking down IRF1 or suppressing IRF1 induction by expressing IRF2 would rescue RelA induced proliferation arrest. Treatment of HRA-kd-IRF1 and HRA-IRF2 cells with Dox according to the scheme in S5C Fig resulted in RelA expression kinetics similar to HRA cells. However, suppressing IRF1 induction by RelA prevented reduction of CDK4 in both HRA-kd-IRF1 and HRA-IRF2 cells. Consequently, there were no changes in p27 and Rb phosphorylation before and after induction of RelA (Fig 6B). Proliferation assays performed using HRA-kd-IRF1 and HRA-IRF2 confirmed that suppressing IRF1 expression rescues RelA induced cell cycle arrest (Fig 6C).

In conclusion, IRF1 is the common down-stream target of RelA and IFN-γ. While RelA induces IRF1 expression directly, IFN-γ induces IRF1 through STAT1 homo-dimers [74]. Increasing RelA levels or stimulation with IFN-γ establishes a positive regulatory circuit between IRF1 and STAT1 that instigate an interferon response resulting in down-regulation of CDK4, up-regulation of p27, suppression of Rb phosphorylation and arresting cells at the G1/S checkpoint. IRF2 inhibits the ability of RelA to induce IRF1 (Fig 6D).

### RelA levels vary widely in breast cancer and negatively correlate with proliferation in certain subtypes

Basal activity of RelA induces an interferon response through IRF1 which downregulates CDK4 and may inhibit CDK2 through up-regulation of p27, resulting in suppression of proliferation. CDK4/cyclin D1 activity is essential for proliferation in the murine mammary epithelium, skin and emergence of tumors driven by HER2/neu consistent with our results obtained in HMEC [52, 53, 75]. Therefore, the RelA-IRF1-CDK4 axis is tumor-suppressive and must be defeated in rapidly growing tumors. Among breast tumors, ER+ tumors display a range of proliferation rates and usually retain a functional Rb. Therefore we hypothesized that ER+ tumors with high levels of RelA are likely to be restrained by the tumor-suppressive RelA-IRF1-CDK4 axis while RelA expression levels may have little impact on the other subtypes of breast tumors.

**Fig 7:**
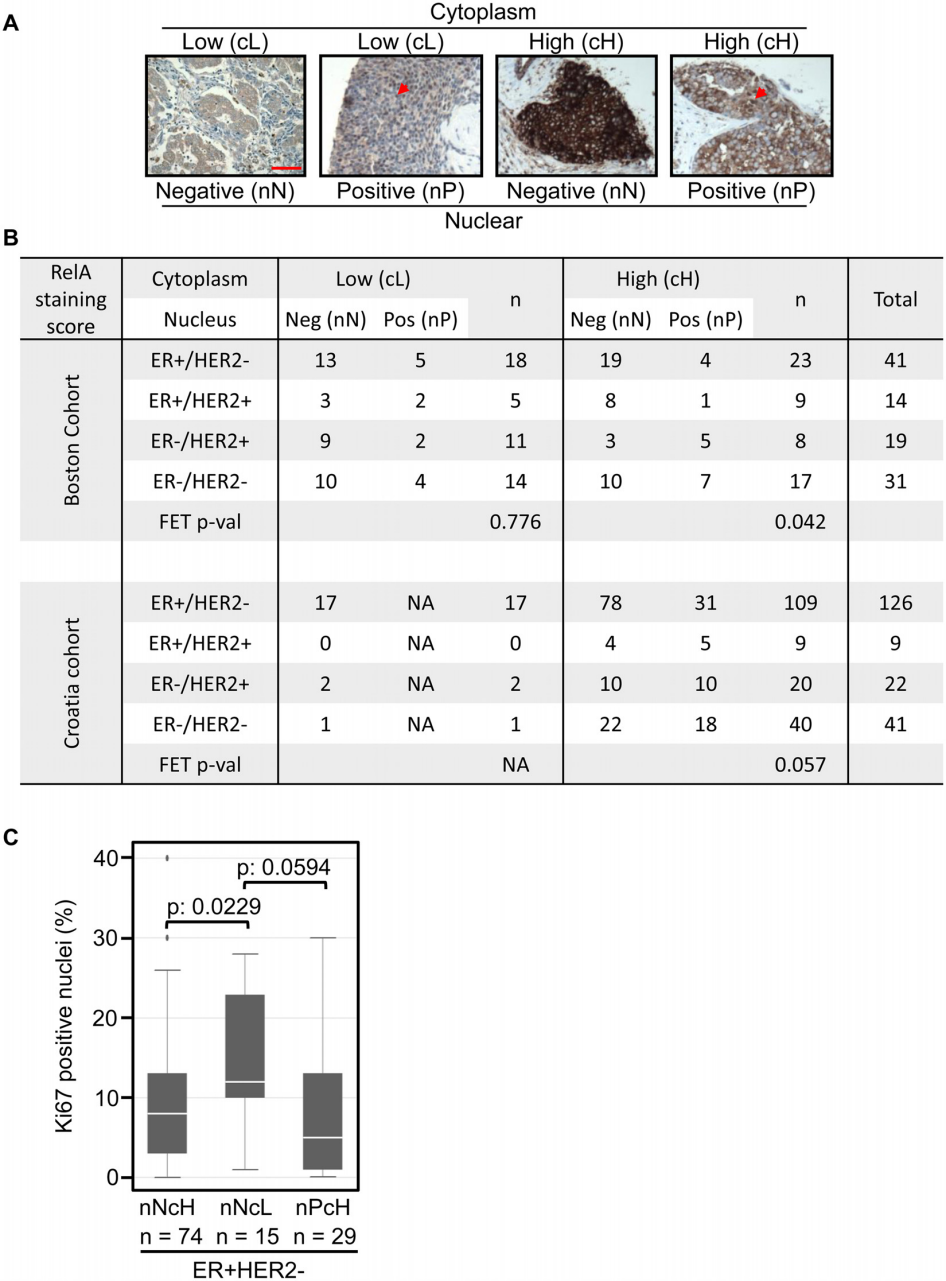
RelA levels vary widely in breast cancer and negatively correlate with proliferation in certain subtypes. **A.** Representative images of breast tumors with each RelA staining profile: cytoplasm low/high; nuclear positive/negative. Red arrows indicate positive nuclei and scale bar = 100μM. **B.** The table shows number of tumors (divided by ER and HER2 based subtypes) within each category of RelA staining pattern in the Boston-cohort (n = 105) and the Croatia-cohort (n = 198). The Fishers exact test (FET) p-values for the distribution is indicated. **C.** Box plot showing the distribution of the percentage of Ki67 positive nuclei within each category of breast tumors based on RelA staining. Nuclear negative/cytoplasm high (nNcH), nuclear negative/cytoplasm low (nNcL) and nuclear positive/cytoplasm high (nPcH). The Wilcoxon rank sum test was used to estimate the significance of the association and the p-values are indicated.

To determine RelA expression levels and activation status in clinical samples, we developed a semi-quantitative IHC method for detecting the intra-cellular distribution of RelA in formalin-fixed paraffin-embedded (FFPE) tissue (S6A Fig and see experimental methods). We stained an exploratory tissue microarray (TMA) containing 105 primary breast tumors (Boston cohort) belonging to four major subtypes of breast cancer. The optimized antibody titer used for staining distinguished a range of RelA levels (from barely detectable to very strong) in the cytoplasm and unambiguously detected nuclear RelA. The majority of tumors contained no or rare nuclear positive (nP) tumor cells within the TMA cores; only few breast tumors harbor >10% nP tumor cells. Based on the intensity of cytoplasmic staining and percentage of nuclear positive tumor cells, tumors were classified into four groups: Nuclear-Negative/Cytoplasm-Low (nNcL); Nuclear-Positive/Cytoplasm-Low (nPcL); Nuclear-Negative/Cytoplasm-High (nNcH) and Nuclear-Positive/Cytoplasm-High (nPcH) and representative cases are shown (Fig 7A). Membership in the four RelA-defined groups was observed in all biomarker-defined subtypes of breast tumors (Boston cohort; Fig 7B and S6B Fig). The distribution of tumors with cytoplasm-low (cL) and cytoplasm-high (cH) RelA staining was approximately equal across biomarker-defined subtypes. Percentage of tumors with nuclear positivity (nP) tends to be higher in ER- tumors (HER2+ and triple-negative tumors) as previously described [16, 76].

Looking at cytoplasmic and nuclear levels in combination, higher levels of cytoplasmic RelA leads to higher nuclear RelA in ER- tumors while this relationship was not observed in ER+ tumors. When cytoplasmic levels were low, percentage of tumors with nuclear RelA was equivalent across subtypes. A prominent feature was the high levels of RelA in ER+ tumors that was confined to the cytoplasm without evidence of activation (19/23), which may be explained by the antagonism between estrogen signaling and NF-kB [77]: ER+ tumors with activated RelA may carry amplification of *IKBKB* or *IKBKE* kinases, or deletion of CYLD (S1 Fig) [35, 78].

Proliferation rates vary widely among ER+ tumors. To evaluate the correlation between proliferation and RelA, we reanalyzed a cohort of breast tumors where the same RelA antibody was used for staining and Ki67 staining was available (Croatia cohort; [44]). The dynamic range of RelA staining in this cohort was lower since a higher concentration of the antibody was used (Croatia cohort; Fig 7B and S6C Fig). Despite this limitation, overall trends were consistent and included the predominance of the nuclear-negative/cytoplasm-high (nNcH) class among ER+ samples, and the tendency to find nuclear positive tumors (nP) among the ER- samples. Furthermore, ER+ tumors with very low RelA levels in both cohorts were identified.

We tested the correlation between RelA levels and proliferative fraction measured by Ki67. Cytoplasmic RelA levels had a significant impact on the proliferation rate of ER+/HER2- tumors (nNcH vs nNcL; p-value 0.0229; Fig 7C). The trend toward lower proliferation in tumors with high cytoplasmic RelA was also observed in tumors with nuclear RelA staining (nNcL vs nPcH; p-value 0.0594; Fig 7C). Proliferation rates in the other subtypes of breast tumors were fairly similar and independent of RelA levels (S6D Fig). These findings validate the tumor-suppressive RelA-IRF1-CDK4 axis within the ER+/HER2- subtype of breast tumors.

## Discussion

RelA is a widely-expressed, pleiotropic NF-kB factor with major roles in development and tissue maintenance [2]. Intriguingly, RelA activity is associated with opposing pro- proliferative oncogenic and anti- proliferative tumor-suppressive roles. We used immortalized primary human mammary epithelial cells (HMEC) to define roles of NF-kB in mammary epithelium and suggest consequences of its activity in breast tumors. These cells have defined requirements for growth and the conditional expression system minimized untraceable effects inherent to constitutive expression systems.

Although RelA is generally thought of as a pro-proliferative factor, we found that expression of RelA diminishes proliferation of HMEC, induces a G1/S cell-cycle block and completely abolishes colony formation in a mammosphere assay. NF-kB Rel proteins with a trans-activating domain negatively regulate proliferation, albeit in specific cell types: RelA in murine fibroblasts, pro-B cells and breast cancer cells, RelB in murine fibroblasts and cRel in HELA cells [18–21, 79]. RelB mediated inhibition of proliferation in mouse embryonic fibroblasts was dependent on upregulation of p53 [20]. Proliferation arrest by cRel in HeLA cells was attributed to increase in H2O2 resulting from upregulation of MnSOD, a target gene of cRel [79]. RelA^-/-^ MEF exhibit genomic aberrations and proliferate faster compared to WT-MEF [21]. Sequestering HDAC was identified as a mechanism driving RelA dependent proliferation arrest in pro-B cells [18]. Although the above mechanisms may operate in HMEC cells expressing higher levels of RelA, our systematic and unbiased approach identified the RelA-IRF1-CDK4 axis as the predominant mediator of RelA dependent suppression of proliferation.

Suppression of proliferation by RelA in HMEC is independent of p53 status, but is dependent upon normal Rb function. The effect is also seen in normal fallopian tube epithelial cells. Amongst the NF-kB Rel proteins, RelB and c-Rel, are both incapable of retarding proliferation in HMEC. Within the time frame we tested, elevation of RelA levels suppressed proliferation while there was no evidence of apoptosis. Furthermore, RelA-induced proliferation arrest was reversible. Upon RelA induction, CDK4 levels drop, p27 levels increase, and Rb protein becomes hypo-phosphorylated. CDK4 activity is crucial for proliferation and development of the murine mammary gland and formation of HER2/neu driven mammary tumors [52, 53]. It is likely that oncogenic transformation of human mammary cells also depends on CDK4. Genetic data from HMEC expressing RelA confirm down-regulation of genes with E2F transcription factor motifs, and implicate interferon response factors as potential intermediates between RelA and the core cell-cycle genes. Mechanistic experiments implicated IRF1 in the proliferation arrest seen after RelA induction. These result suggest a growth suppressive pathway, initiated by RelA-induced IRF1 which induces an interferon response resulting in suppression of CDK4 and a G1/S block in cell-cycle progression.

Analysis of IRF1 in diverse cell types, in-vivo models and tumors indicate tumor-suppressor activity [65]. IRF1^-/-^ mouse embryonic fibroblasts do not senesce upon expression of RasV12 [65]. In the mouse mammary gland, IRF1 regulates the timing of apoptosis during involution [80]. IRF1 expression in ER+ breast tumors negatively correlates with tumor grade [81]. Expression of IRF1 or induction of IRF1 by IFN-γ in breast cancer cell lines suppresses proliferation, induces apoptosis and inhibits xenograft tumor formation of breast cancer cell lines [63, 82]. IRF1 expression is lost in endocrine resistant breast cancer cell lines, and introduction of IRF1 sensitizes the cells to endocrine therapy and apoptosis [57]. Although IRF1 is associated with induction of apoptosis in breast cancer cell lines, expression of IRF1 in non-malignant murine cells did not cause apoptosis [68]. In HMEC, increasing RelA levels induced the expression of BCL2A1 and BIRC3, both anti-apoptotic proteins besides induction of IRF1. Both BCL2A1 and BIRC3 are targets of RelA and upregulation of these genes may help cells resist apoptosis and enter a quiescent state due to reduction in CDK4 and Rb hypo-phosphorylation [72]. In melanoma, BCL2A1 is implicated in resistance to BRAF inhibitors further supporting its pro-survival activity [83].

Since tumor proliferation rate is the major determinant of survival among patients with ER+ disease we sought evidence for the growth suppressive RelA-IRF1-CDK4 axis by analyzing expression profiles of NFKBIA (IkB-α), a surrogate for RelA activity, AURKA, and the interferon factors in ER+/HER2- tumors analyzed by TCGA. Similar to the findings in HMEC, RelA activity positively correlates with expression of IRF1, IRF7 STAT1 and STAT2, negatively correlates with CDK4 and CDK2, and proliferation. It is likely that RelA-dependent suppression of proliferation in a subset of ER+/HER2- tumors is dependent on downregulation of CDK4 by RelA-induced IRF1 and interferon signaling. In fact, high IRF1 expression is significantly associated with longer survival in patients with breast cancer, specifically ER+ disease [84].

A second IRF, IRF2, is thought to antagonize IRF1 transcriptional activity, and is considered a putative oncogene [85]. IRF1 expression is reduced in high-grade breast cancer while it is expressed in normal breast tissue. In contrast, IRF2 is more frequently expressed in high-grade breast tumors, and rarely seen in normal breast tissue [86]. In HRA cells, expression of IRF2 rescued RelA-induced proliferation arrest by suppressing the induction of IRF1. This may be due to the direct binding of RelA and IRF2 and inhibition of IRF1 induction by RelA [87].

Activities of ER, NF-kB and IRF1 determine antiestrogen sensitivity of ER+ breast cancer cells [57]. Inhibiting ER signaling by antiestrogens increases IRF1 expression presumably by releasing ER mediated block of NF-kB. Therefore, differential induction of anti-apoptotic genes directly by NF-kB and pro-apoptotic genes through NF-kB target IRF1, may determine ER+ breast tumor predisposition to succumb or resist endocrine therapy. In breast cancer, NF-kB activation has been measured by nuclear accumulation of RelA in tumor cells. It is widely believed that NF-kB activation, and nuclear localization of RelA, is common in estrogen receptor-negative (ER-) breast cancers. In contrast, NF-kB activation in ER-positive cancers is thought to be uncommon and perhaps specifically inhibited by estrogen signaling through ER [76, 77]. ER, expression of HER2 (erbB2/neu) and histological grade sub classify breast cancers into at least four molecular subtypes [10]. We examined RelA expression in sections of breast cancer tissue from a cohort of tumors that contained examples of each major subtype of disease. In this study, RelA expression was separately reported in the nuclear and cytoplasmic compartments of tumor cells, qualitatively determined by immunohistochemistry and ordinary microscopy. RelA is restrained in the cytoplasm by IkB proteins when NF-kB activation is suppressed, and is released to translocate to the nucleus when NF-kB is activated. The system is self-regulated by potent RelA induction of negative regulators like IkB-α, whose levels rise, pull RelA back into the cytoplasm, and turn down the NF-kB response. We reasoned by recording both the cytoplasmic and nuclear content of RelA, we might gain a more accurate impression of NF-kB activation in the tumor.

In tumors where nuclear RelA is low and cytoplasmic levels high (nPcH), NF-kB activity may be actively restrained, such as in ER-positive tumors without the added stimulus of HER2 (ER+/HER2- subtype). Indeed, 19 of 41 ER+/HER2- tumors (46%) were in the nNcH category of RelA expression. Many other ER+/HER2- cancers expressed no nuclear and little cytoplasm RelA (nNcL); these tumors are perhaps independent of NF-kB influence. ER- cancers are either HER2+ or HER2-. The ER-/HER2- tumors generally do not express the progesterone receptor (PR), which is an ER regulated gene. These tumors are called triple-negative, reflecting lack of expression of ER, PR and HER2. Many of these ER- cancers harbored nuclear RelA (18 of 50, or 36%) and would be deemed NF-kB active. Some of these cancers with evidence of NF-kB activation expressed abundant RelA, which was present in both the nucleus and cytoplasm (nPcH); these cancers may possess an active NF-kB pathway stimulated by paracrine or autocrine factors. Other tumors expressed low levels of RelA, but maintained a prominent fraction of cells with nuclear RelA (nPcL); these cancers may harbor mutations in NF-kB pathway genes. Comparing our data with findings from the TCGA samples, it is likely that these tumors have amplifications of IKBKB or IKBKE or deletions in CYLD (~15% of the samples). The degradation rate of activated, nuclear RelA may be higher in such tumors which leads to low total levels of RelA [88]. However, a number of ER+ cancers did harbor nuclear RelA (12 of 55, or 22%) in the Boston cohort of tumors. Since transcription factors are major determinants of cell-fate, the approach described here can be generalized to other transcription factors and diseases [89].

Proliferation rate is an important determinant of therapy outcomes in patients with ER+ breast tumors. Therefore, we analyzed the correlation between proliferation and RelA-based subtypes within the ER+/HER2- tumor samples in the Croatia-cohort of breast tumors. To our surprise, we found a negative correlation between RelA levels and proliferation. Nuclear positivity has a marginal positive effect on proliferation rate. This observation was consistent in the TCGA dataset. Our results from mechanistic studies in HMEC, gene expression analysis of ER+/HER2- tumors from the TCGA cohort and analysis of ER+/HER2- breast tumors by IHC implicate basal activity of RelA in suppressing proliferation through upregulation of IRF1 and downregulation of CDK4.

Cross-talk between NF-kB and interferon pathways is a component of the innate cellular response to viral infection. Early after infection, interferons and RelA are induced and appear to cooperate in suppressing the cell-cycle, and altering cellular metabolism. Later, cytokine release by infected cells leads to an adaptive immune response, and resolution of the infection [74]. The induction of IRF1 by RelA may be a primitive and pivotal event in response to infection. The RelA to IRF signaling pathway may also originate a robust tumor-suppressor pathway, which may be defeated in many progressively growing cancers and possibly contributing to escape from tumor immune surveillance. Viruses have naturally evolved to defeat this growth suppressive pathway by expression of oncogenic proteins or proteins that inactivate the growth suppression response. A recent genome wide analysis of host cell targets of viral proteins uncovered both NF-kB factors and IRFs in support of the tumor-suppressor activity of this pathway [90].

Palbociclib (PD0332991), an oral small-molecule inhibitor of CDK4/6, is approved for use in combination with the aromatase inhibitor Letrozole in patients with advanced ER+ breast cancers [55]. In the laboratory, only cell lines with functional Rb maintain sensitivity to CDK4/6 inhibition [91, 92]. Many ER+ tumors maintain a functional Rb pathway, while high-grade ER- cancers more frequently inactivate Rb. A practical consequence of our study is the suggestion that strategies to induce RelA or IRF1 activity may also be useful in patients with Rb-competent cancers [93]. Interferon gamma might be a useful agent to provoke the interferon response, and could be used in combination with CDK4/6 inhibitors and hormone therapy.

## Supporting Information

S1 Fig All subtypes of breast tumors carry alterations in the core NF-kB genes. A. The figure was generated in CbioPortal [10, 94, 95]. Survey of copy number alterations and mutation calls generated by sequencing for the indicated genes (in rows) in the TCGA breast cancer dataset (samples in columns). Tumor samples were classified using PAM50 and the sub-types are indicated on top except for one normal-like sample (purple). Estrogen receptor status; Positive (red) Negative (blue) and HER2 status; Positive (yellow), Negative (blue), equivocal (red) and unknown (gray) are indicated. Copy number gain (red bards), copy number loss (blue bars) and mutation (green blocks) are indicated. The genes were divided into three groups: proximal kinases that directly activate the pathway (top group), the core members of the NF-kB family (middle group) and the feedback inhibitors of the pathway (bottom group). The relationship between the groups; activation (green arrow) and inhibition (red arrow) is indicated. Bcl3, NFKBIZ although ankyrin repeat containing proteins, may trans-activate gene expression [96]. IKBKB (IKK-β), IKBKE (IKK-ε) both activators and CYLD a negative regulator, are the most prominent altered genes [35, 78, 97, 98]. In this dataset alterations in NF-kB pathway genes occurs across sub-types in 26% of samples (126/482).
(TIF)

S2 Fig Conditional expression of RelA causes proliferation arrest in epithelial cells. **A.** Induction of apoptosis was monitored in HRA cells over a 60 hour period after induction with Dox (1μg/ml). Whole cell lysates were analyzed by immunoblot using an anti-PARP antibody. **B.** HRA cells were switched to supplement-free medium (SM) for 12 hours and stimulated with fresh SM, full medium (FM) of SM containing EGF (10ng/ml) or Insulin (INS, 10mg/ml) for 15 minutes. Following stimulation, the cells were transferred to ice and whole cell lysates were analyzed by immunoblot using phosphor-specific antibodies to ERK and AKT. **C.** Stable HRA cells constitutively expressing SV40 small T antigen (HRA-st) were generated. HRA and HRA-st cells were plated in triplicates and cultured in the presence or absence of Dox (1μg/ml) for 3 days and the amount of cells under each condition was estimated using the MTS assay.
(TIF)

S3 Fig RelA induced proliferation arrest is Rb dependent. **A.** Sequence of the oligonucleotide, and its salient features, used to convert the Tetracycline regulated expression plasmid pRXTN for expressing miR-shRNAs is shown. **B.** Box depicting the range of tumor purity within the TCGA cohort of breast tumors classified based on clinical markers ER and HER2. Fraction of tumors cells within each sample (Tumor purity) was obtained from ESTIMATE database [42]. **C.** Correlation between expression of AURKA and RelA in ER+/HER2- breast tumors from the TCGA cohort where the tumor fraction in the sample was estimated to be > 75%.
(TIF)

S4 Fig RelA induction down-regulates CDK4 resulting in Rb hypo-phosphorylation and cell cycle arrest. **A.** Schematic representation of the protocol used to generate triplicate samples for gene expression analysis. All samples (ND 1–3; 24+ 1–3, 72+ 1–3 and DW 1–3) were plated 12 hours prior to time 0 (indicated at the bottom). Empty bars indicate absence of Dox and filled bars indicate presence of Dox. Black arrows indicate addition of Dox to the media, green arrow indicates withdrawal of Dox and red arrow indicates processing of sample for RNA extraction. Medium in all samples was changed every 24 hours with required (-/+ Dox) containing medium. **B.** Venn diagram shows the number of genes up or down-regulated compared to the ND sample and comparison to the other conditions. The Venn diagram was generated using a web tool [99]. **C.** Schematic representation of the experimental protocol used to analyze reversibility of RelA induced proliferation arrest by immunoblot. The scheme is similar to **A** except that all samples were harvested after 72 hours. **D.** Bar plot showing log2 expression values of pro- and anti-apoptotic genes identified to be significantly (FDR < 0.05) differentially expressed in the ND, 24+, 72+ and DW samples.
(TIF)

S5 Fig RelA induced interferon response may be responsible of CDK4 down-regulation and proliferation arrest. **A.** The bar plot shows log2 expression values of the Type I–Type III receptors and ligands in ND, 24+, 72+ and DW samples of HRA cells. **B.** IRF1 is a known target of RelA and its promoter contains multiple RelA-NF-kB binding motifs. This analysis was performed using RVista 2.0 [100].
(TIF)

S6 Fig High RelA correlates with diminished proliferation in breast cancer subtypes. **A.** FFPE sections of SKOV3 cells unstimulated or stimulated with TNF-α for 15 minutes were stained using the optimized RelA staining protocol. **B and C.** Distribution of breast tumors in the Boston and Croatia cohorts within RelA-based subtypes expressed as percentage of tumors within each breast cancer subtype. This is an alternative representation of the table in Fig 7B. **D.** Box plots showing the distribution of tumors based on RelA-based subtypes and percentage of Ki67-positive nuclei for ER+/HER+, ER-/HER2+ and ER-/HER2- breast cancer subtypes. These distributions were statistically insignificant.
(TIF)
