# Supplementary figures and images for: RelA-Induced Interferon Response Negatively Regulates Proliferation
Source: PLoS One. 2015 Oct 13;10(10):e0140243. doi: 10.1371/journal.pone.0140243 (PMC4604146; doi:10.1371/journal.pone.0140243)

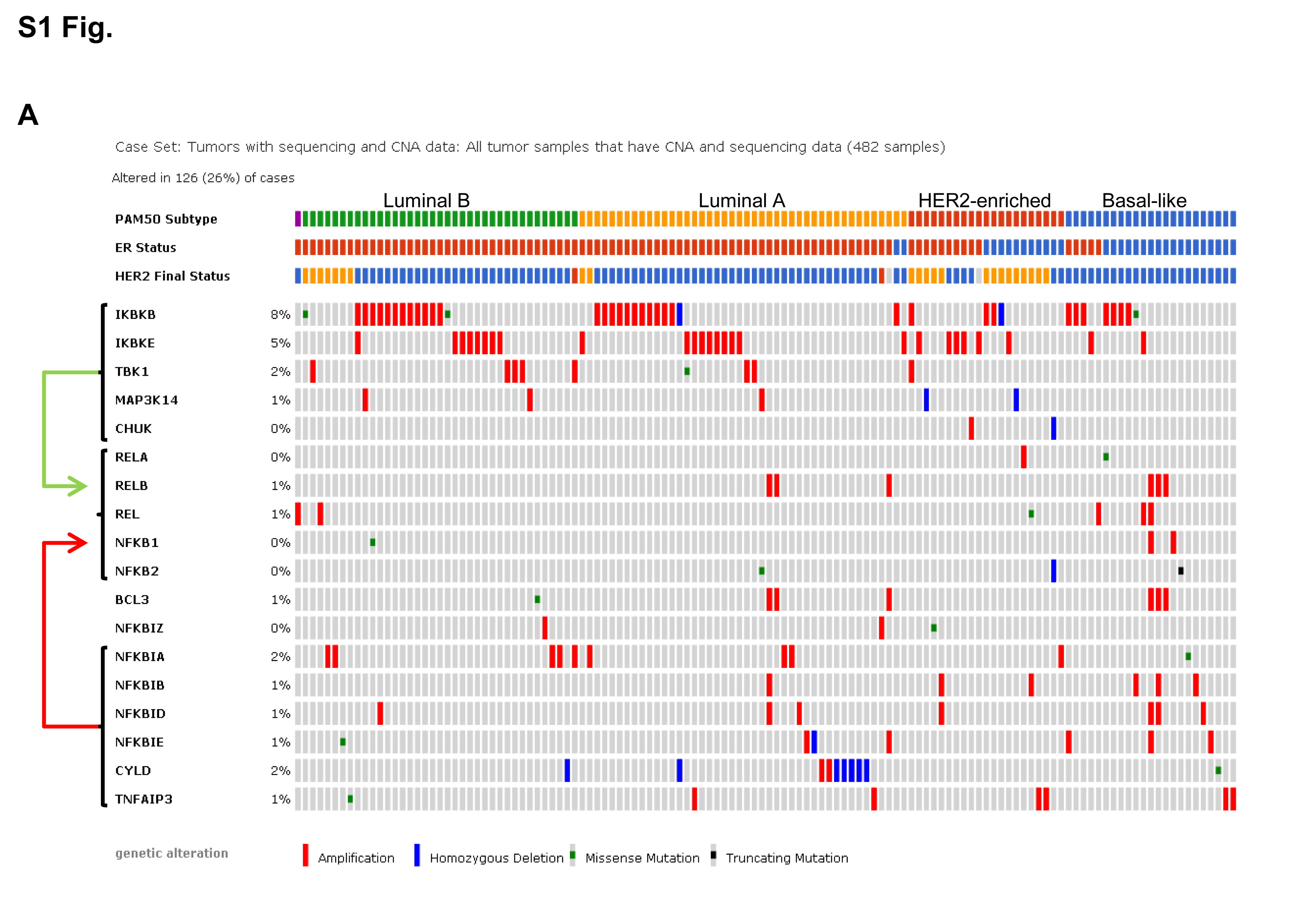

Supplement: S1 Fig — A. The figure was generated in CbioPortal [10, 94, 95]. Survey of copy number alterations and mutation calls generated by sequencing for the indicated genes (in rows) in the TCGA breast cancer dataset (samples in columns). Tumor samples were classified using PAM50 and the sub-types are indicated on top except for one normal-like sample (purple). Estrogen receptor status; Positive (red) Negative (blue) and HER2 status; Positive (yellow), Negative (blue), equivocal (red) and unknown (gray) are indicated. Copy number gain (red bards), copy number loss (blue bars) and mutation (green blocks) are indicated. The genes were divided into three groups: proximal kinases that directly activate the pathway (top group), the core members of the NF-kB family (middle group) and the feedback inhibitors of the pathway (bottom group). The relationship between the groups; activation (green arrow) and inhibition (red arrow) is indicated. Bcl3, NFKBIZ although ankyrin repeat containing proteins, may trans-activate gene expression [96]. IKBKB (IKK-β), IKBKE (IKK-ε) both activators and CYLD a negative regulator, are the most prominent altered genes [35, 78, 97, 98]. In this dataset alterations in NF-kB pathway genes occurs across sub-types in 26% of samples (126/482). (TIF) [file pone.0140243.s001.tif]

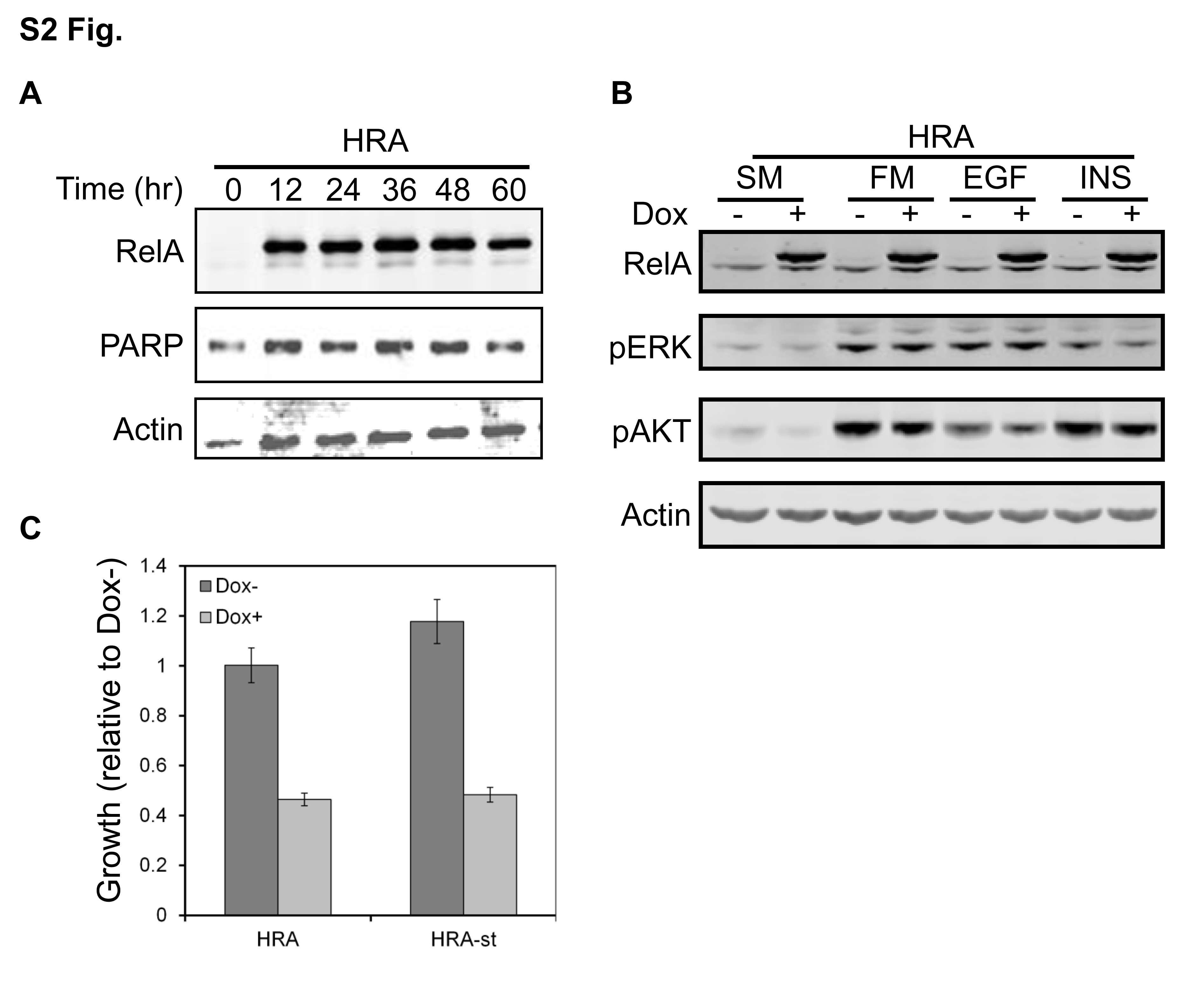

Supplement: S2 Fig — A. Induction of apoptosis was monitored in HRA cells over a 60 hour period after induction with Dox (1μg/ml). Whole cell lysates were analyzed by immunoblot using an anti-PARP antibody. B. HRA cells were switched to supplement-free medium (SM) for 12 hours and stimulated with fresh SM, full medium (FM) of SM containing EGF (10ng/ml) or Insulin (INS, 10mg/ml) for 15 minutes. Following stimulation, the cells were transferred to ice and whole cell lysates were analyzed by immunoblot using phosphor-specific antibodies to ERK and AKT. C. Stable HRA cells constitutively expressing SV40 small T antigen (HRA-st) were generated. HRA and HRA-st cells were plated in triplicates and cultured in the presence or absence of Dox (1μg/ml) for 3 days and the amount of cells under each condition was estimated using the MTS assay. (TIF) [file pone.0140243.s002.tif]

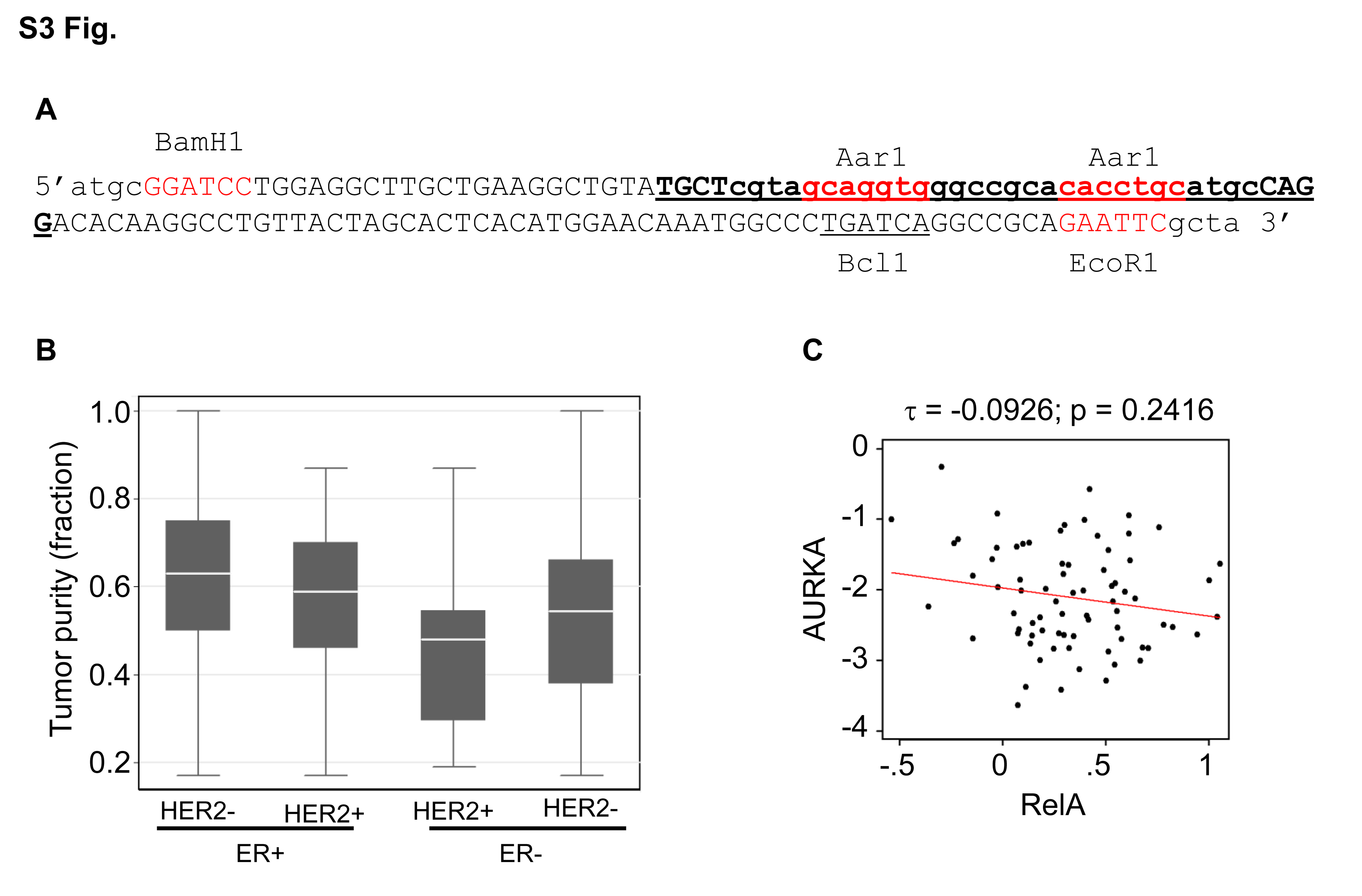

Supplement: S3 Fig — A. Sequence of the oligonucleotide, and its salient features, used to convert the Tetracycline regulated expression plasmid pRXTN for expressing miR-shRNAs is shown. B. Box depicting the range of tumor purity within the TCGA cohort of breast tumors classified based on clinical markers ER and HER2. Fraction of tumors cells within each sample (Tumor purity) was obtained from ESTIMATE database [42]. C. Correlation between expression of AURKA and RelA in ER+/HER2- breast tumors from the TCGA cohort where the tumor fraction in the sample was estimated to be > 75%. (TIF) [file pone.0140243.s003.tif]

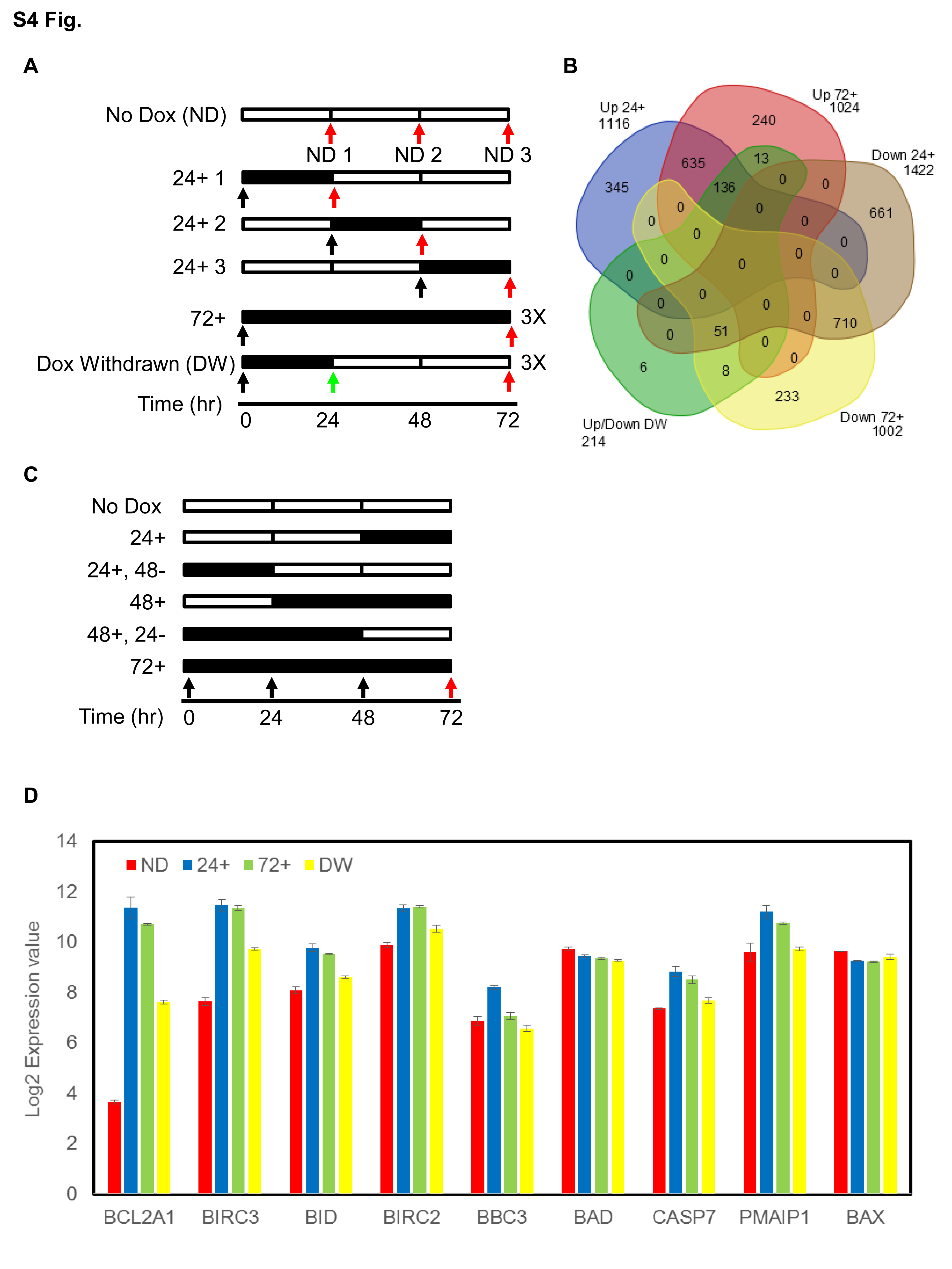

Supplement: S4 Fig — A. Schematic representation of the protocol used to generate triplicate samples for gene expression analysis. All samples (ND 1–3; 24+ 1–3, 72+ 1–3 and DW 1–3) were plated 12 hours prior to time 0 (indicated at the bottom). Empty bars indicate absence of Dox and filled bars indicate presence of Dox. Black arrows indicate addition of Dox to the media, green arrow indicates withdrawal of Dox and red arrow indicates processing of sample for RNA extraction. Medium in all samples was changed every 24 hours with required (-/+ Dox) containing medium. B. Venn diagram shows the number of genes up or down-regulated compared to the ND sample and comparison to the other conditions. The Venn diagram was generated using a web tool [99]. C. Schematic representation of the experimental protocol used to analyze reversibility of RelA induced proliferation arrest by immunoblot. The scheme is similar to A except that all samples were harvested after 72 hours. D. Bar plot showing log2 expression values of pro- and anti-apoptotic genes identified to be significantly (FDR < 0.05) differentially expressed in the ND, 24+, 72+ and DW samples. (TIF) [file pone.0140243.s004.tif]

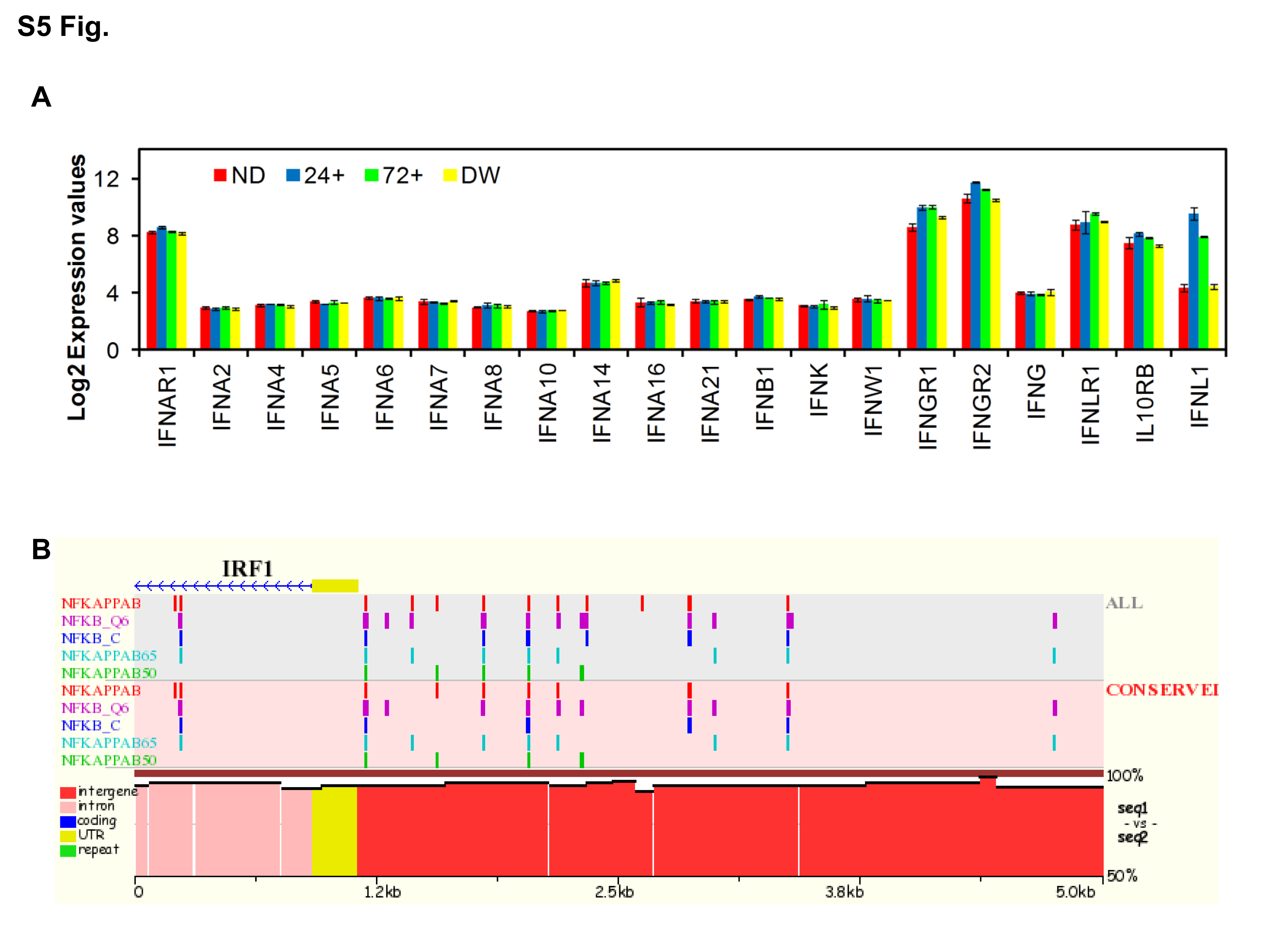

Supplement: S5 Fig — A. The bar plot shows log2 expression values of the Type I–Type III receptors and ligands in ND, 24+, 72+ and DW samples of HRA cells. B. IRF1 is a known target of RelA and its promoter contains multiple RelA-NF-kB binding motifs. This analysis was performed using RVista 2.0 [100]. (TIF) [file pone.0140243.s005.tif]

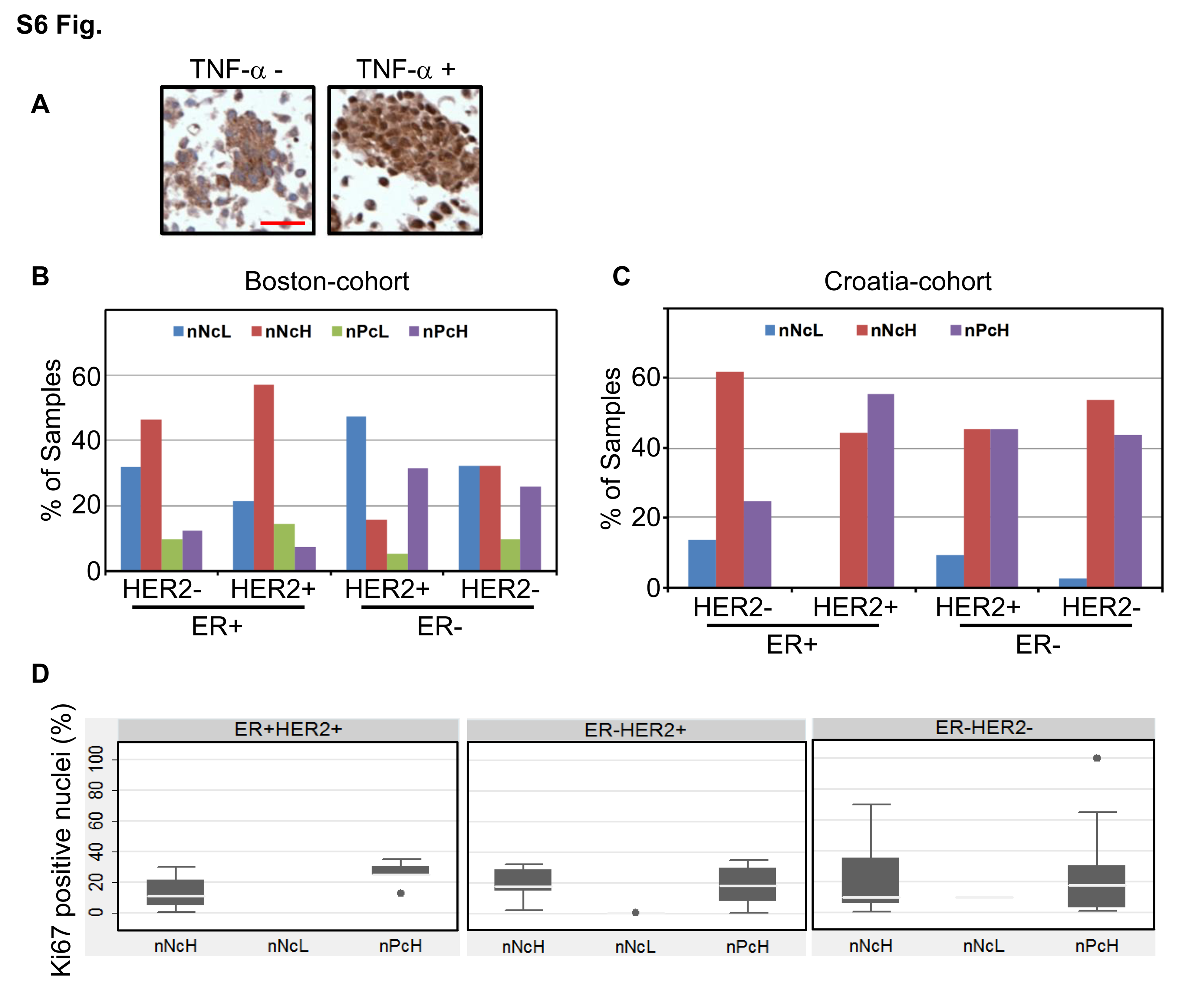

Supplement: S6 Fig — A. FFPE sections of SKOV3 cells unstimulated or stimulated with TNF-α for 15 minutes were stained using the optimized RelA staining protocol. B and C. Distribution of breast tumors in the Boston and Croatia cohorts within RelA-based subtypes expressed as percentage of tumors within each breast cancer subtype. This is an alternative representation of the table in Fig 7B. D. Box plots showing the distribution of tumors based on RelA-based subtypes and percentage of Ki67-positive nuclei for ER+/HER+, ER-/HER2+ and ER-/HER2- breast cancer subtypes. These distributions were statistically insignificant. (TIF) [file pone.0140243.s006.tif]
